# Aqueous bio-based hydrotropic solutions for green extraction of glycyrrhizic acid from licorice: experimental investigation and mechanistic insights

**DOI:** 10.1039/d6ra02244h

**Published:** 2026-04-21

**Authors:** Nhan Trong Le, Hang Thanh Thi Phan, The-Huan Tran, Hung The Nguyen, Hoai Thi Nguyen

**Affiliations:** a Hue University of Medicine and Pharmacy, Hue University Hue City Vietnam nthoai@hueuni.edu.vn; b Hanoi University of Pharmacy Hanoi City Vietnam

## Abstract

Aqueous bio-based hydrotropic solutions (ABHS) were investigated as green solvents for extracting glycyrrhizic acid (GA), a major amphiphilic triterpenoid saponin from licorice. Conventional and bio-based hydrotropes were screened under ultrasound-assisted extraction, with pentane-1,2-diol (PED) identified as the most effective solvent. Under optimized conditions (67.9% ABHS concentration, liquid–solid ratio of 49.9 mL g^−1^, extraction time of 17.0 min, and extraction temperature of 70 °C), ABHS achieved high GA extraction yield of 79.43 mg g^−1^, markedly outperforming conventional aqueous alcohols (≈49 mg g^−1^) and reported eutectic solvents (45.47–61.29 mg g^−1^). Extraction kinetics followed a second-order model, with a low apparent activation energy of 19.84 kJ mol^−1^, indicating a diffusion-controlled extraction process. Quantum chemical analyses, including electrostatic potential mapping, interaction region indicator analysis, and binding energy evaluation, revealed cooperative stabilization of both polar glycosidic units and weakly polar triterpenoid domains of GA in ABHS, with a calculated binding energy of −19.798 kcal mol^−1^, significantly stronger than those observed for water (−12.137 kcal mol^−1^) and ethanol (−15.307 kcal mol^−1^). An integrated macroporous resin-based strategy enabled efficient GA recovery with a recovery efficiency of 92.98% and high GA content (80.31%), while maintaining stable extraction performance and solvent recovery above 95.50% over three reuse cycles. Furthermore, GA-enriched fraction obtained using ABHS exhibited enhanced nitric oxide inhibitory activity without detectable cytotoxic effects on normal human cells, as supported by molecular docking analysis. In this context, the present study establishes ABHS as efficient, sustainable, and mechanistically transparent platforms for GA extraction, offering a promising green strategy for amphiphilic triterpenoid saponins.

## Abstract 2

Aqueous bio-based hydrotropic solutions enable efficient and recyclable extraction of glycyrrhizic acid from licorice with enhanced bioactivity.

## Graphical abstract

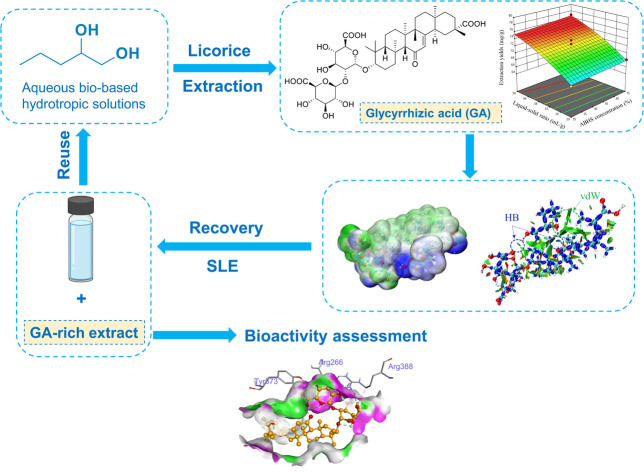

## 1. Introduction

Licorice (*Glycyrrhiza uralensis* Fisch.) is one of the most extensively used medicinal plants in both traditional and modern medicine, owing to its broad spectrum of pharmacological activities, including anti-inflammatory, antiviral, hepatoprotective, and immunomodulatory effects.^1,2^ Among its diverse bioactive constituents, glycyrrhizic acid (GA), a triterpenoid saponin, is widely recognized as the principal marker compound responsible for many of the therapeutic properties of licorice.^3,4^ Structurally, GA exhibits a pronounced amphiphilic character, comprising highly polar glycosidic moieties linked to a weakly polar triterpenoid aglycone.^3^ This structural duality plays a crucial role in governing its solubility behavior, biological activity, and extractability from plant matrices.

Conventional extraction of GA commonly employs aqueous alcohols, particularly ethanol (EtOH) or methanol (MeOH) based systems,^4,5^ owing to their suitable polarity and ease of implementation. While these solvents can achieve satisfactory extraction yields, their performance often relies on relatively large solvent volumes or extended extraction conditions to ensure efficient recovery of GA from plant matrices.^5^ Moreover, the use of volatile organic solvents raises increasing concerns regarding environmental sustainability, solvent recovery, and occupational safety, particularly in the context of large scale processing. These limitations have stimulated growing interest in the development of greener and more sustainable solvent systems capable of improving extraction efficiency while reducing environmental impact.^6,7^

In recent years, various green solvent platforms have been investigated for the extraction of GA in an effort to overcome the limitations associated with conventional solvents.^4,5,8–12^ Deep eutectic solvents (DESs) and natural DESs have attracted considerable attention due to their low volatility, tunable polarity, and strong hydrogen bonding capability.^13–15^ Several studies have demonstrated that acid based or polyol based DESs can enhance GA solubilization compared with water or aqueous alcohols, particularly when the solvent acidity or polarity is well matched with the physicochemical properties of GA. Assisted extraction techniques, including ultrasound and microwave irradiation, have further been combined with DESs to improve mass transfer and reduce extraction time.^8,10^ Moreover, downstream strategies such as macroporous resins (MRs) have been explored to facilitate GA recovery from DESs extracts.^12^

Despite these advances, the practical application of DESs for GA extraction remains limited by several inherent constraints. High viscosity, multicomponent formulation complexity, and challenges associated with solvent regeneration can hinder mass transfer, solvent handling, and process scalability, particularly in the extraction of amphiphilic molecules such as GA.^16^ In many reported studies, the extraction performance of GA using DESs has been evaluated primarily at an empirical level, while the mechanistic basis of solvent–solute interactions, kinetic control, and structure-dependent extraction behavior has remained insufficiently elucidated.^4,5,8–12^ These limitations highlight the need for alternative green solvent systems that combine efficient solubilization of GA with simpler composition, lower viscosity, and improved mechanistic transparency.

Hydrotropes constitute a unique class of small amphiphilic molecules that improve the dissolution of sparingly soluble substances in water through the formation of dynamic, non micellar aggregation domains. Unlike surfactants, hydrotropes do not form stable micelles and typically operate without a well-defined critical micelle concentration, allowing effective solubilization across a broad concentration range.^17,18^ These characteristics make hydrotropes particularly suitable for improving the solubilization of structurally complex molecules containing both polar and hydrophobic domains, such as amphiphilic natural products including GA. Classical hydrotropes, such as sodium salicylate, sodium citrate, urea, sodium benzenesulfonate, and sodium benzoate, have long been employed to improve the solubility of poorly soluble organic compounds in aqueous systems, and have found applications in pharmaceutical and chemical processing.^17,19^

More recently, increasing attention has been directed toward the development of bio-based hydrotropes derived from renewable resources, including alkanediols, γ-valerolactone, cyrene, ethyl lactate, and glycerol ethers.^7^ In addition to retaining the characteristic solubilization behavior of conventional hydrotropes, this class of compounds offers distinct advantages associated with their bio-based origin, such as derivation from biomass feedstocks, improved biodegradability, reduced toxicity, and enhanced environmental compatibility.^20^ These bio-based hydrotropes therefore combine favorable sustainability profiles with effective solubilization capability arising from cooperative hydrogen-bonding and hydrophobic interactions. Several studies have demonstrated that aqueous solutions of such bio-based solvents, commonly referred to as aqueous bio-based hydrotropic solutions (ABHS), exhibit significant potential for enhancing the solubility and extraction efficiency of natural products.^20–24^

When mixed with water, bio-based hydrotropes create tunable solvent microenvironments that facilitate hydrogen bonding with polar sites while simultaneously enabling hydrophobic association with nonpolar regions of solute molecules.^18^ This dual interaction mechanism renders ABHS particularly suitable for the extraction of amphiphilic compounds such as GA, whose efficient solubilization requires cooperative stabilization of both polar glycosidic units and weakly polar triterpenoid domains. More broadly, triterpenoid saponins share similar amphiphilic structural features,^25^ suggesting that ABHS may represent a versatile platform for their solubilization. Nevertheless, the applicability of ABHS to triterpenoid saponin recovery has not yet been systematically established, and experimental evidence elucidating their extraction behavior and solvent–solute interaction mechanisms remains scarce.

In this study, a comprehensive investigation was conducted to evaluate ABHS as green solvents for the extraction of GA from licorice. A series of conventional hydrotropes and ABHS were initially screened under ultrasound-assisted conditions to identify efficient solvent candidates. Selected ABHS were subsequently optimized to elucidate the effects of key process parameters. Extraction kinetics were analyzed to clarify rate-controlling mechanisms and temperature dependence. To gain molecular-level insight into solvent–solute interactions, quantum chemical calculations, including electrostatic potential (ESP) mapping, interaction region indicator (IRI) analysis, and binding energy evaluation, were employed. Furthermore, an integrated recovery strategy based on MRs was developed to enable efficient enrichment, solvent regeneration, and overall process sustainability. The anti-inflammatory activity and cytotoxicity of GA-enriched fraction obtained using ABHS were finally evaluated and correlated with molecular docking analysis, thereby establishing ABHS as efficient, sustainable, and mechanistically well-understood platforms for the extraction of GA and related amphiphilic natural products.

## 2. Materials and methods

### 2.1. Plant materials and chemical reagents

Licorice roots were obtained from a specialized medicinal herbs company in Hue City, Vietnam, and authenticated based on standard pharmacognostic criteria in accordance with the Vietnamese Pharmacopoeia.^26^ A voucher specimen (no. LCR-001) was deposited at the Faculty of Pharmacy, University of Medicine and Pharmacy, Hue University, Vietnam. The plant material was air-dried at an appropriate temperature, ground into a fine powder, and stored under dry and dark conditions until further use. GA standard was purchased from Macklin Inc. (Guangzhou, China). Traditional hydrotropes and ABHS listed in Table 1 were obtained from Xilong Scientific and Macklin Inc. (Guangzhou, China). Lipopolysaccharides from *Escherichia coli* were supplied by Sigma Chemical Co. (Missouri, USA). The murine macrophage cell line RAW 264.7 was provided by the University of Perugia (Italy), while human embryonic kidney cells (HEK-293A, RRID: CVCL_0045) were kindly provided by Prof. Chi-Ying F. Huang, the National Yang Ming Chiao Tung University (Taiwan). Dulbecco's Modified Eagle Medium and fetal bovine serum were sourced from Thermo Fisher Scientific (Massachusetts, USA). MRs and other chemicals used in the extraction, recovery, analytical, and biological assays were obtained from reputable commercial suppliers and were of analytical or HPLC grade. Deionized water was produced using a water-purification system (Avidity Science, USA). Ultrasound-assisted extraction was performed using an ultrasonic bath operating at 37 kHz and 150 W (Elma, Germany). Quantification of GA in the extracts was conducted using reversed-phase HPLC (Agilent 1260 Infinity II, USA). The solvent was removed and the extract was concentrated with a rotary evaporator (Büchi R-300, Switzerland). Absorbance measurements in the biological assays were recorded using a microplate reader (BioTek ELx800, BioTek Instruments, USA). Proton Nuclear Magnetic Resonance (^1^H NMR) spectra were recorded on a Bruker Avance NEO 600 spectrometer (Bruker, USA), while the chemical composition of the samples was further characterized by UHPLC-Q-TOF-MS/MS employing an ExionLC™ UHPLC system coupled to an X500R QTOF mass spectrometer (AB SCIEX, USA).

**Table 1:** Types, compositions, concentrations, and abbreviations of traditional hydrotropes and ABHS used for solvent screening

Type	Solvents	Abbreviation
Traditional hydrotropes	Sodium salicylate 25%	SS 25%
	Sodium salicylate 50%	SS 50%
	Sodium citrate 25%	SC 25%
	Urea 25%	UR 25%
	Urea 50%	UR 50%
	Sodium benzoate 25%	SB 25%
Aqueous bio-based hydrotropic solutions (ABHS)	Ethyl lactate 50%	ETL 50%
	Ethyl lactate 100%	ETL 100%
	γ-Valerolactone 50%	GVL 50%
	γ-Valerolactone 100%	GVL 100%
	Ethane-1,2-diol 50%	ETD 50%
	Ethane-1,2-diol 100%	ETD 100%
	Propane-1,2-diol 50%	PRD 50%
	Propane-1,2-diol 100%	PRD 100%
	Butane-1,2-diol 50%	BUD 50%
	Butane-1,2-diol 100%	BUD 100%
	Pentane-1,2-diol 50%	PED 50%
	Pentane-1,2-diol 100%	PED 100%
	Hexane-1,2-diol 50%	HED 50%
	Hexane-1,2-diol 100%	HED 100%

### 2.2. Solvent preparation and screening by ultrasound-assisted extraction

Traditional hydrotropes and ABHS were prepared by dissolving the corresponding components in deionized water at predefined concentrations. The mixtures were magnetically stirred until complete dissolution was achieved, yielding homogeneous and stable systems prior to extraction. The compositions, concentrations, and abbreviations of the investigated solvents are summarized in Table 1. Solvent screening was conducted using ultrasound-assisted extraction to evaluate the extraction performance of the prepared hydrotropic systems toward GA. A fixed amount of licorice powder was mixed with each hydrotropic solvent at a liquid-to-solid ratio of 20 mL g^−1^. The extraction was carried out in an ultrasonic bath at 50 °C for 30 min to ensure consistent and comparable conditions across all solvent systems. After extraction, the mixtures were centrifuged and filtered to obtain clear supernatants. The resulting extracts were stored at low temperature prior to subsequent analysis.

### 2.3. Experimental design and optimization

A response surface methodology (RSM) framework implemented through a Box–Behnken design (BBD) was employed to optimize the ultrasound-assisted extraction of GA using PED ABHS. Four independent variables, including ABHS concentration, liquid–solid ratio, extraction time, and extraction temperature, were evaluated at three coded levels (−1, 0, +1), with their actual values shown in Table S1. The extraction yield of GA obtained using PED was selected as the response variable. Experimental runs were performed according to the BBD matrix and the data were fitted to a second-order polynomial model. Model adequacy was assessed by analysis of variance, and response surface plots were used to identify the optimal extraction conditions.

### 2.4. Kinetic modeling of GA extraction

The kinetics of GA extraction using PED was analyzed to clarify the time dependent extraction behavior and the influence of temperature on mass transfer. The experimental data were interpreted using a second order kinetic model, which assumes that the extraction rate depends on the square of the difference between the equilibrium yield and the yield at a given extraction time. Accordingly, the extraction rate can be expressed as d*C*_*t*_/d*t* = *k*(*C*_s_ − *C*_*t*_)^2^. Integration of this equation under the initial condition *C*_*t*_ = 0 at *t* = 0 results in a linear relationship between *t*/*C*_*t*_ and *t*, expressed as *t*/*C*_*t*_ = *t*/*C*_s_ + 1/(*kC*_s_^2^). The initial extraction rate was calculated from the kinetic parameters as *h* = *kC*_s_^2^. To evaluate the effect of temperature on the extraction kinetics, the rate constants obtained at different temperatures were correlated using the Arrhenius equation, *k* = *A*_e_ exp(−*E*_a_/*RT*). The apparent activation energy was determined from the slope of the linear plot of ln *k versus* 1/*T*. In these equations, *k* (g mg^−1^ min^−1^) is the second order kinetic rate constant; *C*_*t*_ and *C*_s_ (mg g^−1^) denote the extraction yields at time *t* and at equilibrium, respectively; *t* represents the extraction time (min); *h* (mg g^−1^ min^−1^) is the initial extraction rate; *E*_a_ (kJ mol^−1^) is the apparent activation energy; *A*_e_ (mg g^−1^ min^−1^) is the Arrhenius pre exponential factor; *R* is the universal gas constant (8.314 J mol^−1^ K^−1^); and *T* is the absolute temperature (K).

### 2.5. Computational methods

#### 2.5.1. Quantum chemical calculations

Electronic structure calculations were used to probe the interaction features between GA and selected solvent molecules using a simplified 1 : 1 solute–solvent complex model. All computations were performed with the ORCA 6.1.0 package.^27–30^ Molecular geometries of GA and the selected solvents (PED, EtOH, and H_2_O), along with their corresponding GA–solvent complexes (GA–PED, GA–EtOH, and GA–H_2_O), were constructed in Avogadro^31^ and subsequently pre-optimized using the MMFF94 force field. Subsequent geometry optimizations were performed in the gas phase using density functional theory at the B3LYP-D3(BJ)/def2-SVP level of theory, where D3(BJ) denotes Grimme's dispersion correction with Becke–Johnson damping. The RIJCOSX approximation was employed to accelerate the calculations, together with TightSCF convergence criteria. All reported energies were obtained at the same level of theory as the geometry optimizations and used consistently for comparative analysis. The resulting optimized structures were analyzed to obtain ESP distributions and IRI descriptors^32^ using Multiwfn 3.8,^33,34^ with molecular visualizations produced in VMD 1.9.4.^35^ Interaction strengths were quantified through binding energy calculations according to: Δ*E*_binding_ = *E*_complex_ − (*E*_solute_ + *E*_solvent_), where more negative values correspond to stronger solute–solvent affinity.

#### 2.5.2. Molecular docking method

Molecular docking was conducted using AutoDock Vina^36^ to evaluate the binding affinity of GA toward inducible nitric oxide synthase (iNOS, PDB: 3E7G).^37^ The preparation of the protein and ligand structures followed the procedure described in our previous work.^38^ Docking was carried out using a grid box covering the catalytic cavity near the heme cofactor, with an exhaustiveness level of 16. The top-ranked pose was analyzed in ChimeraX^39^ and BIOVIA Discovery Studio to characterize key interactions between GA and active-site residues of iNOS.

### 2.6. Recovery of GA using MRs

The recovery of GA from ABHS extracts was performed using a solid–liquid extraction (SLE) approach combined with MRs.^20^ Briefly, 10 g of MRs were packed into columns of identical dimensions and sequentially conditioned with absolute EtOH, alkaline solution, and acidic solution, followed by thorough rinsing with deionized water until neutral conditions were achieved to stabilize the adsorption surface. Extracts obtained under optimized extraction conditions were then loaded onto the columns at a fixed volume. During the separation process, the ABHS phase was first eluted with deionized water, while GA was selectively retained on the MRs matrix. Subsequently, the target compound was desorbed sequentially using 50% EtOH and absolute EtOH. The collected fractions were concentrated under reduced pressure to obtain GA-enriched products for subsequent analyses. The selection and performance of MRs were evaluated based on two parameters: GA recovery (%) and GA content (%), calculated as GA recovery = (*m*_1_/*m*_0_) × 100% and GA content = (*m*_1_/*m*_2_) × 100%, respectively. Here, *m*_0_ represents the mass of GA present in the extract prior to recovery, *m*_1_ denotes the mass of GA recovered in the desorbed fractions, and *m*_2_ corresponds to the mass of the GA-enriched extract obtained after solvent removal.

### 2.7. Recycling of ABHS and MRs

Recycling of the ABHS was carried out by concentrating the aqueous eluates to remove excess water, followed by adjustment of the ABHS concentration to the optimal level for subsequent extraction cycles. In parallel, MRs were regenerated after each cycle through sequential treatment with alkaline and acidic solutions, followed by thorough washing with deionized water until neutral pH was achieved, and then reused in the following recovery cycles. The efficiency and operational stability of the integrated extraction–recovery process were evaluated over successive cycles. Four parameters were employed to assess the recycling performance, including extraction yield, GA recovery, GA content, and ABHS recovery.

### 2.8. HPLC quantification of GA

Quantification of GA was performed following a previously reported method by Kavita *et al.*^5^ with minor modifications. Analyses were conducted using a reversed-phase HPLC system equipped with a diode array detector and an autosampler. Chromatographic separation was achieved on an Eclipse XBD-C18 column (4.6 × 250 mm, 5.0 µm, Agilent Technologies), with the column temperature maintained at 30 °C. The mobile phase consisted of MeOH and 0.1% aqueous trifluoroacetic acid at a volumetric ratio of 7 : 3. The flow rate was set at 0.5 mL min^−1^, and detection was carried out at 254 nm. Representative chromatograms of the standard and sample solutions are shown in Fig. S1. A linear calibration model (*y* = 6.5322*x* + 50.505, *R*^2^ = 0.9998) was applied for GA quantification (Fig. S2), where *x* and *y* correspond to GA concentration (µg mL^−1^) and peak area (mAU s), respectively.

### 2.9. Nitric oxide inhibitory activity

The nitric oxide inhibitory activity was evaluated using lipopolysaccharide (LPS)-stimulated RAW 264.7 macrophages following reported methods with minor modifications.^40^ Cells were seeded in 96-well plates (2 × 10^6^ cells per mL) and incubated for 24 h, then pretreated with different concentrations of the samples for 2 h prior to stimulation with LPS (1 µg mL^−1^) for an additional 24 h. Dexamethasone was used as the positive control. Nitric oxide production was determined by measuring nitrite levels in the culture supernatant using the Griess reaction, with absorbance recorded at 540 nm. Nitrite concentrations were calculated from a sodium nitrite standard curve, and NO inhibition was expressed relative to the LPS-treated control. Cell viability was simultaneously assessed using the MTT assay, and IC_50_ values were determined by nonlinear regression analysis.

### 2.10. Cytotoxicity assay on HEK-293A cells

The cytotoxicity of the tested samples was evaluated on human embryonic kidney cells (HEK-293A) using the sulforhodamine B (SRB) assay with minor modifications.^41^ Cells were seeded in 96-well plates and allowed to attach for 18–20 h, followed by treatment with serial dilutions of the samples prepared in serum-free medium, with the final dimethyl sulfoxide concentration not exceeding 0.5%. After 48 h of incubation, cells were fixed with cold trichloroacetic acid, stained with SRB solution, and washed to remove unbound dye. The protein-bound dye was solubilized in unbuffered Tris base, and absorbance was measured at 540 nm using a microplate reader. Ellipticine was used as the positive control, while vehicle-treated cells served as the negative control. Cell growth inhibition was calculated relative to the control, and IC_50_ values were determined by nonlinear regression analysis.

### 2.11. Statistical analysis

Data are reported as mean ± standard deviation from three independent experiments. Statistical evaluation was performed using analysis of variance followed by the least significant difference test (*p* < 0.05), while extraction optimization was conducted in Design-Expert (version 13.0) within a response surface framework.

## 3. Results and discussion

### 3.1. Screening of ABHS for GA extraction

The rationale for employing ABHS for the extraction of GA is based on several key considerations. (i) From a structural perspective, GA exhibits a pronounced amphiphilic character, consisting of highly polar glycosidic units and a weakly polar triterpenoid backbone.^3^ This structural feature closely matches the amphiphilic nature of ABHS,^18^ which enables simultaneous interactions with both hydrophilic and hydrophobic domains, thereby facilitating GA solubilization and enhancing extraction efficiency in aqueous media. (ii) ABHS have demonstrated considerable potential for the extraction of natural products;^20^ however, comprehensive investigations and in-depth mechanistic understanding of hydrotrope-assisted extraction processes remain limited. (iii) From a solvent design and process engineering standpoint, ABHS represent simplified solvent systems with minimal component complexity compared to multicomponent platforms such as DESs.^5,8^ This simplification not only strengthens the green profile of the extraction process but also favors solvent recovery, recyclability, and economic feasibility, which are critical considerations for process scale-up and industrial implementation while maintaining high extraction performance.

Following the above considerations, an initial solvent screening was conducted to evaluate the extraction performance of GA from licorice. All investigated solvents were employed in aqueous form, including traditional hydrotropes and ABHS. Detailed information on the investigated solvents is provided in Table 1. Conventional solvents, namely water, MeOH, and EtOH, were also included as reference solvents. All extractions were performed under identical experimental conditions to ensure a reliable comparison. The obtained extraction yields exhibited a pronounced dependence on solvent composition and hydrotropic characteristics.

**Fig. 1:**
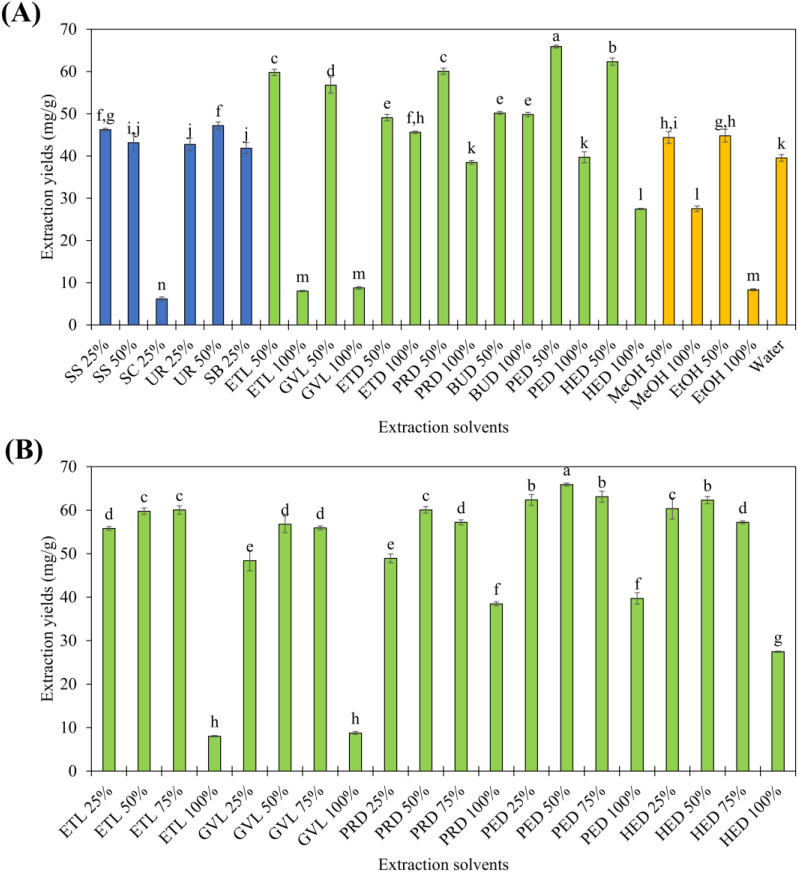
Extraction yields of GA from licorice. (A) Initial solvent screening using traditional hydrotropes, ABHS, and reference solvents. (B) Effect of solvent concentration on GA extraction yields for selected ABHS. Different letters indicate statistically significant differences among means (*p* < 0.05).

The initial screening results are summarized in Fig. 1A. Traditional hydrotropes afforded GA extraction yields ranging from 6.17 to 47.20 mg g^−1^, whereas ABHS showed a broader performance window, with extraction yields ranging from 8.03 to 65.91 mg g^−1^. Among the investigated systems, several ABHS, including GVL 50%, PED 50%, and HED 50%, exhibited markedly higher GA extraction yields than water alone, confirming the effectiveness of hydrotropic-assisted solubilization. In contrast, neat ABHSs, such as ETL 100%, GVL 100%, PED 100%, and HED 100%, generally resulted in reduced extraction efficiency. This behavior suggests that excessive organic content or insufficient water disrupts effective hydrotropic interactions, thereby limiting GA solubilization.

Based on the initial screening, five ABHS exhibiting relatively high extraction performance were selected for further evaluation at different aqueous concentrations (25%, 50%, 75%, and 100%) to identify the optimal solvent composition. These systems included ETL, GVL, PRD, PED, and HED, and the corresponding results are presented in Fig. 1B. Distinct concentration-dependent extraction behaviors were observed for each hydrotrope. Notably, PED 50% consistently delivered the highest GA extraction yield within the intermediate concentration range, reaching values of 65.91 mg g^−1^. The extraction capacity achieved using PED 50% was competitive with those obtained using reference solvents, which exhibited extraction yields in the range of 8.32–44.79 mg g^−1^. Based on their superior extraction performance and favorable concentration-dependent profiles, PED was identified as the most promising ABHS for subsequent process optimization and mechanistic investigations.

The variation in GA extraction efficiency observed among different ABHS can be primarily interpreted in terms of structural compatibility between the solvent system and the target compound. GA is an amphiphilic molecule comprising highly polar glycosidic units and a weakly polar triterpenoid backbone.^3^ ABHS similarly exhibit amphiphilic characteristics, with distinct hydrophilic and hydrophobic domains.^18^ This structural similarity enables ABHS to interact concurrently with both domains of GA, thereby providing a favorable environment for hydrotrope-assisted solubilization.

From a molecular design perspective, the length of the hydrophobic carbon chain in ABHS plays a critical role in determining extraction performance. As the carbon chain length (ETD, PRD) increases from short to medium-chain diols (PED, HED, ETL, and GVL), GA extraction efficiency improves accordingly. This enhancement reflects stronger van der Waals interactions between the hydrophobic segments of the ABHS and the triterpenoid core of GA, while the presence of hydroxyl groups preserves sufficient hydrophilicity to maintain solubility in aqueous media. Such a balance is essential for effective hydrotropic solubilization.

Notably, among the investigated alkanediols, PED exhibited the highest extraction efficiency, suggesting the existence of an optimal structural balance within this solvent class. Compared to shorter-chain diols, PED provides enhanced hydrophobic interaction with the triterpenoid backbone of GA due to its intermediate carbon chain length. At the same time, the presence of two hydroxyl groups ensures sufficient polarity to maintain strong hydrogen bonding interactions with the glycosidic moieties and surrounding water molecules. In contrast, shorter-chain diols tend to exhibit weaker hydrophobic interactions, while longer-chain counterparts such as HED may display increased viscosity and enhanced hydrophobicity, which can reduce effective polarity, hinder solvent–water interactions, and ultimately limit mass transfer and solvent accessibility. Therefore, PED appears to offer an optimal balance between hydrophobic and hydrophilic interactions, resulting in its superior extraction performance.

Solvent concentration further governs the extraction behavior of GA. At low to moderate aqueous concentrations, ABHS molecules participate in hydrotrope-driven solvation,^20^ in which their hydrophobic regions preferentially associate with the triterpenoid backbone of GA, while their polar functional groups interact extensively with surrounding water molecules. In this regime, the polar glycosidic moieties of GA are stabilized through hydrogen bonding with water, whereas the hydrophobic core is accommodated *via* solvent-guided interactions with the ABHS, resulting in an efficient and cooperative solvation environment.

In contrast, at high hydrotrope concentrations or in neat solvent systems, GA extraction efficiency decreases markedly. Experimentally, the extraction efficiencies obtained using all ABHS at 100% concentration were consistently lower than those achieved at 50% concentration. This decline can be attributed to multiple factors, including disruption of cooperative hydrotropic interactions, reduction of the structural role of water, and a pronounced increase in solvent viscosity.^20^ Elevated viscosity hampers molecular diffusion and reduces mass transfer rates, thereby limiting the transport of GA from the plant matrix into the solvent phase. Under such conditions, a transition toward water-driven or solvent-dominated solvation may occur,^22^ resulting in an unfavorable solvation environment for GA and consequently diminished extraction performance.

Beyond their solubilizing capacity, the amphiphilic character of ABHS facilitates the penetration of the solvent system into plant tissues. By reducing interfacial tension, ABHS can enhance wetting and permeation of the plant matrix, facilitating solvent access to intracellular compartments. This behavior is comparable to a mild surfactant effect, which can partially loosen plant cell wall structures and facilitate the liberation of GA from intracellular compartments, thereby improving extraction efficiency.^20,24^

Collectively, these findings indicate that GA extraction efficiency is not governed by solvent strength alone but is determined by the formation of a balanced hydrotropic environment that simultaneously optimizes solubilization, mass transfer, and matrix penetration. Within this framework, PED emerges as an optimal ABHS, as it provides an appropriate balance between hydrophobic interactions with the triterpenoid backbone of GA and hydrogen-bond stabilization of the glycosidic moieties through the aqueous network, while maintaining sufficiently low viscosity to facilitate efficient mass transfer. Moreover, the predominantly noncovalent and reversible nature of hydrotropic interactions offers additional advantages for downstream recovery and solvent recycling. This combination accounts for their superior extraction performance and provides a sound mechanistic basis for their selection in subsequent process optimization and molecular-level investigations.

### 3.2. Extraction optimization

To systematically evaluate the effects of process variables on the extraction efficiency of GA from licorice, RSM combined with a BBD was employed. Four independent variables, including ABHS concentration, liquid–solid ratio, extraction time, and extraction temperature, were selected, while GA extraction efficiency was considered as the dependent response. The experimental matrix consisting of 29 runs designed according to the BBD, along with the corresponding GA extraction efficiencies, is presented in Table S2.

The ANOVA results confirmed that the quadratic regression model established for the PED ABHS was highly significant (Table S3), with a model *p* value below 0.0001. High values of the coefficient of determination (*R*^2^), adjusted *R*^2^, and predicted *R*^2^ (all >0.90), together with a non-significant lack of fit test (*p* = 0.9028), indicate that the proposed model provides a reliable fit to the nonlinear relationships between the process variables and GA extraction efficiency within the studied design space. The close correspondence between experimental and model-predicted values (Fig. S3), together with the low relative deviations summarized in Table S4, further validates the adequacy of the RSM model. These observations substantiate its robustness and predictive reliability for process optimization. The regression equation expressing GA extraction efficiency as a function of the independent variables is provided below:*Y* = 44.42848 + 0.005439*A* + 0.591995*B* + 0.152150*D* − 0.001481*BC* − 0.003403*CD* − 0.003815*B*^2^ − 0.001235*C*^2^In this equation, *Y* represents the GA extraction efficiency obtained using the PED ABHS. *A*, *B*, *C*, and *D* correspond to the independent variables, namely ABHS concentration, liquid–solid ratio, extraction time, and extraction temperature, respectively.

**Fig. 2:**
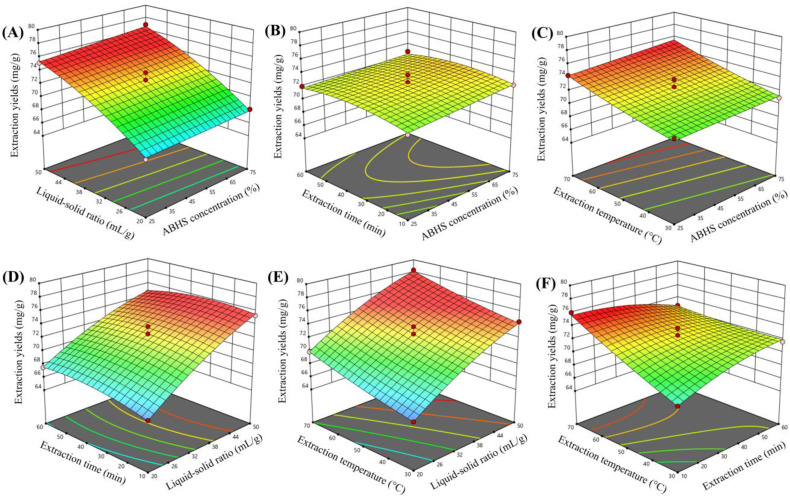
Response surface plots showing the effects and interactions of extraction variables on GA extraction efficiency in PED ABHS. (A) ABHS concentration and liquid–solid ratio; (B) ABHS concentration and extraction time; (C) ABHS concentration and extraction temperature; (D) liquid–solid ratio and extraction time; (E) liquid–solid ratio and extraction temperature; (F) extraction time and extraction temperature.

Considering the individual effects of the investigated variables, the liquid–solid ratio was identified as the most influential factor governing GA extraction efficiency. This conclusion is strongly supported by the exceptionally high *F* value obtained from the ANOVA analysis (*F* = 915.16), accompanied by a highly significant *p* value (*p* < 0.0001) (Table S3). As the liquid–solid ratio increased from low to higher levels, GA extraction efficiency increased markedly due to the enhanced concentration gradient between the solvent and the plant matrix, as well as the improved penetration of the solvent into the plant tissue.^20^ These trends are consistently illustrated by the response surface plots presented in Fig. 2A, D, and E.

The ABHS concentration exhibited a pronounced nonlinear effect on GA extraction efficiency, as reflected in Fig. 2A–C. At low ABHS concentrations, the solvent environment was insufficient to form effective hydrotropic domains capable of interacting with the weakly polar triterpenoid backbone of GA, leading to limited extraction efficiency. Increasing the ABHS concentration to an intermediate range significantly enhanced GA extraction, owing to the synergistic contribution of water and bio-based hydrotropes, which enabled the simultaneous stabilization of the hydrophilic glycosidic moieties and the hydrophobic aglycone segment of GA. In contrast, at high ABHS concentrations, GA extraction efficiency tended to plateau or slightly decrease, which can be attributed to the reduced availability of free water and the increased viscosity of the solvent system, thereby restricting intraparticle diffusion.^20^

The extraction time, when considered as an individual factor, did not exert a statistically significant effect on GA extraction efficiency within the investigated domain, with all corresponding *p* values exceeding 0.05. GA extraction increased predominantly during the initial stage of the process and rapidly reached a steady state, indicating that GA extraction in ABHS proceeds efficiently and approaches equilibrium within a relatively short duration. This behavior is evident in Fig. 2B, D, and F. These results highlight the strong solubilization capability of the hydrotropic environment, which diminishes the role of prolonged diffusion controlled extraction.^20^

The extraction temperature exerted a positive effect on GA extraction efficiency within an intermediate range, as demonstrated by Fig. 2C, E, and F, with *p*-values all lower than 0.0001. The enhancement can be attributed to the reduction in solvent viscosity, the increase in diffusion coefficients, and the facilitation of interactions between GA and the solvent, as well as the disruption of GA matrix binding. However, further increases in temperature did not result in proportional gains in extraction efficiency, reflecting the attainment of solubilization equilibrium and the increased risk of co extraction of undesired components.^20^

In addition to the individual effects of the process variables, GA extraction efficiency was strongly influenced by their combined interactions, as evidenced by the curvature and topology of the response surfaces shown in Fig. 2. Only a limited number of interaction terms were statistically significant. In particular, the interaction between the liquid–solid ratio and extraction time exhibited a significant effect (*p* = 0.0365), along with a much stronger interaction between extraction time and temperature (*p* < 0.0001).

At low liquid–solid ratios, extending the extraction time resulted in only marginal improvements in GA extraction efficiency, owing to insufficient solvent availability to maintain an effective solubilization gradient. As the liquid–solid ratio increased to an intermediate level, the influence of extraction time became more pronounced, reflecting the synergistic interplay between solvent availability and mass transfer. However, at high liquid–solid ratios, the contribution of prolonged extraction time diminished, as the system rapidly approached solubilization equilibrium, as illustrated in Fig. 2D.

The interaction between extraction time and temperature further highlights the kinetic nature of GA extraction in PED ABHS. At intermediate temperatures, increasing the extraction time enhanced GA recovery due to reduced solvent viscosity and improved diffusion. In contrast, when prolonged extraction times were combined with elevated temperatures, no proportional increase in extraction efficiency was observed, indicating that the system had reached equilibrium and that excessive thermal input may compromise extraction selectivity, as reflected in Fig. 2F.

In addition to the linear and interaction effects, the significance of the quadratic terms further confirms the pronounced curvature of the response surfaces and the nonlinear nature of GA extraction in the ABHS. The quadratic terms *B*^2^ (*p* = 0.0005) and *C*^2^ (*p* = 0.0011) were statistically significant (Table S3), indicating the presence of optimal liquid–solid ratios and extraction times, beyond which further increases do not proportionally enhance extraction efficiency due to solubilization saturation and mass transfer limitations.

The optimal extraction conditions were established as follows: an ABHS concentration of 67.9%, a liquid–solid ratio of 49.9 mL g^−1^, an extraction time of 17.0 min, and an extraction temperature of 70 °C. Under these conditions, the GA extraction efficiency reached 79.43 ± 1.13 mg g^−1^. The experimentally obtained value was in close agreement with the model predicted response, confirming the accuracy and reliability of the RSM model for process optimization.

### 3.3. Comparative evaluation of PED in relation to previously reported solvents

**Fig. 3:**
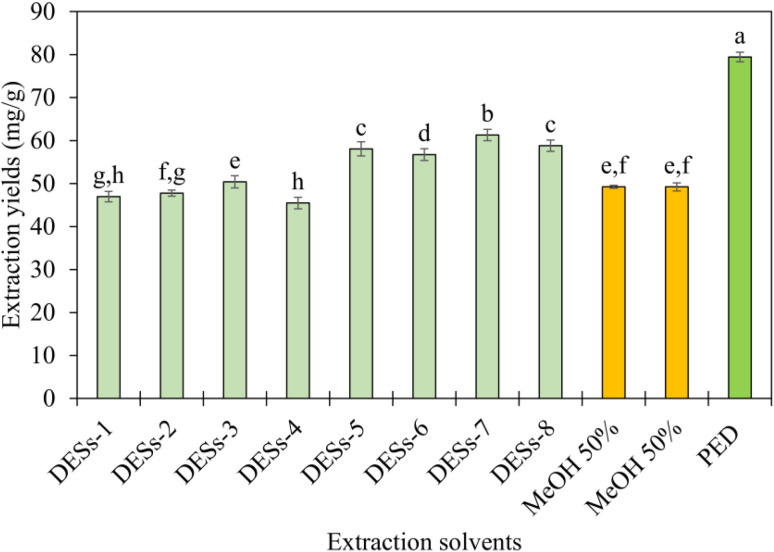
Comparison of GA extraction yields achieved by PED ABHS, green solvents reported in the literature, and commonly used reference solvents. Different letters indicate statistically significant differences among means (*p* < 0.05).

The extraction performance of PED ABHS was systematically evaluated in comparison with a range of green solvents previously reported for GA extraction, particularly deep eutectic solvents (DESs),^5,8–11^ as well as conventional aqueous organic solvents commonly used as reference extraction media. Under the unified experimental framework established in this study, the literature-reported DES systems exhibited GA extraction yields ranging from 45.47 to 61.29 mg g^−1^, whereas the reference aqueous organic solvents (50% MeOH and 50% EtOH) afforded maximum yields of approximately 49 mg g^−1^. In contrast, PED ABHS achieved a markedly higher GA extraction yield of 79.43 mg g^−1^. The extraction conditions applied for each solvent system are summarized in Table S5, while the corresponding extraction yields are presented in Fig. 3.

Direct numerical comparisons between extraction yields reported in independent studies are inherently subject to uncertainty, as variations in plant origin, cultivation conditions, sample processing, and experimental protocols can substantially influence extraction outcomes. In view of these limitations, all solvent systems included in the present comparison, encompassing literature-reported green solvents as well as reference organic solvents, were systematically re-examined using the same batch of plant material under strictly controlled and identical experimental conditions. By re-performing all extraction experiments within a unified laboratory framework, variability arising from inter-study differences was minimized, thereby enabling a more reliable and internally consistent assessment of solvent performance.

The superior performance of PED can be attributed to its distinctive solvent architecture, which generates a tunable hydrotropic microenvironment capable of mediating cooperative hydrogen bonding and hydrophobic interactions. Such synergistic solvation behavior facilitates more effective disruption of plant matrices and promotes selective solubilization of GA across a broader polarity range without requiring harsh extraction conditions.

Collectively, the results of this internally controlled comparative study clearly establish PED as a highly efficient extraction medium for GA when evaluated on an equivalent experimental basis. Beyond its superior extraction efficiency, PED offers additional practical advantages over DESs, as it consists of a single-component solvent rather than a multicomponent eutectic mixture. This structural simplicity reduces formulation complexity, improves economic feasibility, and facilitates solvent recovery and reuse. In addition, compared to DESs that often exhibit high viscosity and require energy-intensive handling and separation steps, PED-based ABHS operate at lower viscosity and enable more efficient mass transfer, which can reduce process energy requirements. From a cost perspective, the use of a single bio-based component with increasing commercial availability further contributes to the feasibility of large-scale implementation. Taken together, these attributes underscore the strong potential of PED as an effective and sustainable solvent platform for the extraction of triterpenoid saponins from medicinal plants.

### 3.4. Extraction mechanism

#### 3.4.1. ESP analysis and binding energy evaluation of GA in different solvent environments

ESP analysis was employed to elucidate the electronic complementarity between GA and different solvent components, thereby providing a molecular-level rationale for the experimentally observed extraction efficiencies. Water and EtOH were selected as reference solvents, as aqueous EtOH (50%) represents the most efficient conventional solvent system for GA extraction, while water constitutes the continuous phase of ABHS. This comparison allows the specific contribution of the hydrotrope, namely PED, to be distinguished from that of water alone.

**Fig. 4:**
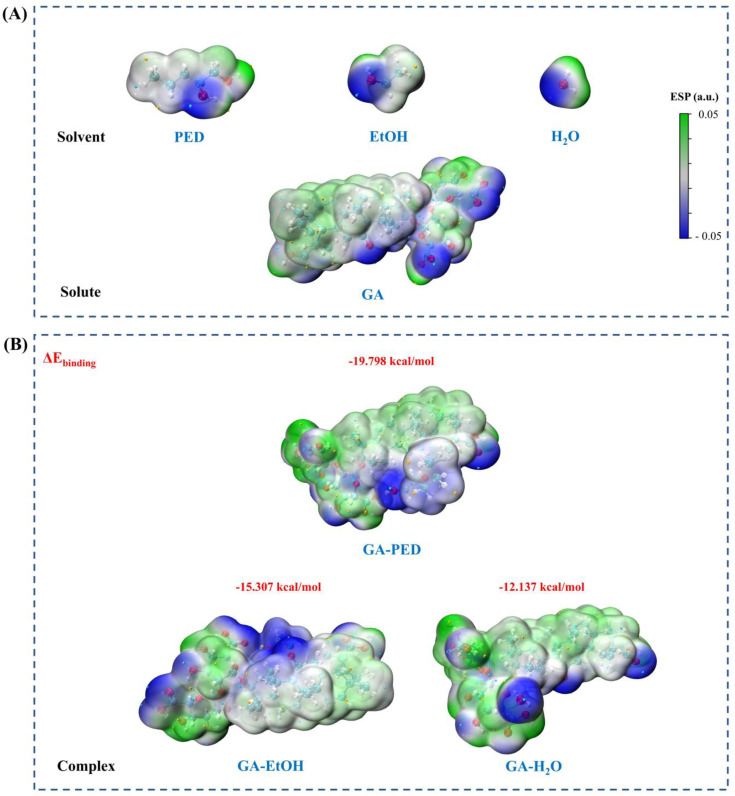
ESP maps and binding energies of GA in different solvent environments. (A) ESP maps of the isolated solvent molecules (PED, EtOH, and H_2_O) and GA. (B) ESP maps of the GA–solvent complexes (GA–PED, GA–EtOH, and GA–H_2_O) with the corresponding binding energies.

As shown in Fig. 4, GA exhibits a highly heterogeneous ESP distribution. Pronounced negative potential regions are predominantly localized on the carboxylate and hydroxyl groups within the glycosidic moiety, whereas the triterpenoid backbone displays largely neutral to slightly positive potential. This spatial separation of polar and nonpolar regions highlights the intrinsically amphiphilic nature of GA, indicating that efficient molecular stabilization requires a solvent environment capable of interacting with both domains simultaneously.

Comparison of GA interactions with the conventional solvents used in this study, namely water and EtOH, reveals relatively limited electronic compatibility. The calculated binding energies for the GA–H_2_O and GA–EtOH systems are −12.137 and −15.307 kcal mol^−1^, respectively. Corresponding ESP maps indicate that electrostatic interactions are primarily localized around the polar functional groups of GA, while large portions of the hydrophobic triterpenoid backbone remain weakly screened. These results suggest that water and EtOH predominantly stabilize GA through localized polar interactions, providing insufficient coverage of its nonpolar surface.

In contrast, markedly stronger electronic interactions are observed for GA in the presence of PED. The binding energy reaches −19.798 kcal mol^−1^ for the GA–PED system. Importantly, the ESP maps reveal a more complementary electrostatic distribution surrounding the GA surface in the presence of PED, indicating improved electronic compatibility between the solvent and the polar regions of GA. This effect arises from the amphiphilic character of PED, which aligns well with the amphiphilic structure of GA, thereby enabling more effective stabilization of the entire molecule.

It should be noted that the calculated binding energies represent relative electronic interaction strengths obtained under identical computational conditions in the gas phase, rather than absolute solvation free energies in bulk solution. Nevertheless, because all solvent systems were evaluated consistently using the same theoretical framework, the observed energy trends provide a reliable comparative basis for assessing solvent–solute interaction preferences. Within this context, the substantially more favorable interaction energy observed for the GA–PED system clearly indicates stronger local electronic interactions relative to those formed with water or EtOH.

Furthermore, the results demonstrate that PED plays a dominant role in governing solvent–solute interactions within the ABHS. Although the system operates in an aqueous medium, the significantly stronger interactions between GA and PED, compared with GA–H_2_O, indicate that water alone cannot account for the observed stabilization. Instead, PED creates a favorable microenvironment that simultaneously accommodates the polar and nonpolar domains of GA. This molecular-level modulation of the electrostatic environment provides a rational explanation for the superior extraction performance of the ABHS demonstrated experimentally.

#### 3.4.2. IRI analysis of noncovalent interactions between GA and different solvents

IRI analysis was conducted to elucidate the nature and spatial distribution of noncovalent interactions governing the stabilization of GA in different solvent environments. Unlike ESP, which reflects overall electronic compatibility, IRI enables direct identification of weak interaction regions such as hydrogen bonding and van der Waals interactions, thereby providing more detailed insight into solvent–solute interaction mechanisms.

**Fig. 5:**
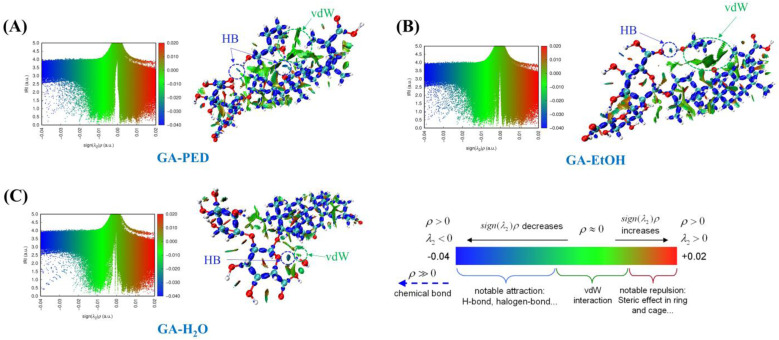
IRI scatter plots and isosurface representations showing the distribution of noncovalent interactions between GA and different solvent systems. (A) GA–PED system, (B) GA–EtOH system, and (C) GA–H_2_O system.

As shown in Fig. 5B and C, the GA–H_2_O system exhibits only limited interaction regions, which are predominantly localized around the carboxylic and hydroxyl groups of the glycosidic moiety and correspond mainly to localized hydrogen bonding interactions. In contrast, the triterpenoid backbone shows almost no discernible interaction regions. For the GA–EtOH system, the IRI surfaces reveal a moderate increase in both the number and extent of interaction regions compared with water, including hydrogen bonding and weak dispersive interactions. Nevertheless, these interactions remain spatially fragmented and are still primarily associated with polar functional groups, with limited coverage of the triterpenoid backbone. The uneven distribution of interaction regions in both systems indicates that water and EtOH preferentially stabilize the polar domains of GA, while their ability to interact effectively with the nonpolar domain remains limited, resulting in incomplete overall stabilization of the molecule.

In contrast, the ABHS represented by the GA–PED system exhibits a pronounced increase in hydrogen bonding along with extensive and more continuous noncovalent interaction regions distributed across the entire GA structure (Fig. 5A). The IRI analysis reveals the presence of hydrogen bonds formed between the diol hydroxyl groups of PED and the carboxylic and hydroxyl groups of GA (O–H⋯O), together with broad van der Waals interaction regions surrounding the nonpolar triterpenoid backbone. This interaction pattern reflects the ability of PED to cooperatively engage both the polar and nonpolar domains of GA.

### 3.5. Extraction kinetics

The extraction kinetics of GA from licorice using the ABHS were investigated to elucidate the effects of extraction time and temperature on both the extraction rate and the extent of GA recovery, thereby providing further insight into the controlling mechanisms of the extraction process. The experimental data were well described by a second order kinetic model using the linearized form of *t*/*C*_*t*_*versus t*, and calculated kinetic parameters are listed in Table 2. The high correlation coefficients obtained for the PED ABHS at all investigated temperatures demonstrate excellent agreement between the experimental data and the second order model, confirming its suitability for describing GA extraction in this system (Fig. S4).

**Table 2:** Second order kinetic parameters describing GA extraction from licorice using the PED ABHS^a^

	*T* (°C)	*C*_s_ (mg g^−1^)	*h* (mg g^−1^ min^−1^)	*k* (g mg^−1^ min^−1^)	*R*_1_^2^	*E*_a_ (kJ mol^−1^)	ln *A*_e_	*R*_2_^2^
PED	40	70.42	238.10	0.0480	0.9999	19.84	4.2481	0.9982
	50	70.42	303.03	0.0611	1.0			
	60	71.94	384.62	0.0743	1.0			

a *k* is the second-order rate constant (g mg^−1^ min^−1−1^), *C*_s_ is the equilibrium GA yield (mg g^−1^), *h* is the initial extraction rate (mg g^−1^ min^−1−1^), *E*_a_ is the activation energy (kJ mol^−1^), *A*_e_ is the Arrhenius pre-exponential factor (mg g^−1^ min^−1−1^), and *T* is the extraction temperature (°C). *R*_1_^2^ and *R*_2_^2^ denote the coefficients of determination corresponding to the second-order kinetic model and the Arrhenius fitting, respectively.

The equilibrium extraction yield (*C*_s_) increased only slightly as the temperature rose from 40 to 60 °C, ranging from 70.42 to 71.94 mg g^−1^. In contrast, the kinetic parameters associated with extraction rate, including the initial extraction rate (*h*) and the rate constant (*k*), increased markedly with temperature. Specifically, *h* increased from 238.10 to 384.62 mg g^−1^ min^−1^ and *k* increased from 0.0480 to 0.0743 g mg^−1^ min^−1^ as the temperature increased from 40 to 60 °C. This trend indicates that elevating the temperature predominantly enhances the extraction kinetics, while exerting a negligible influence on the final equilibrium yield.

Arrhenius analysis based on the relationship between ln *k* and 1/*T* enabled the determination of the apparent activation energy of the GA extraction process (Fig. S5). The *E*_a_ value obtained for the PED ABHS was 19.84 kJ mol^−1^. According to commonly applied kinetic interpretations, *E*_a_ values lower than 20 kJ mol^−1^ are typically associated with diffusion-controlled processes, whereas values between 20 and 40 kJ mol^−1^ suggest mixed diffusion and solubilization control. Based on this criterion, GA extraction in the PED ABHS can be classified as diffusion-controlled.

Altogether, the kinetic results indicate that PED is an effective solvent system for GA extraction, with a diffusion-dominated mechanism characterized by a relatively low energy barrier and rapid attainment of equilibrium. The moderate activation energy suggests that mass transfer plays a predominant role in controlling the extraction process under the investigated conditions. These findings provide mechanistic support for the efficient extraction performance of PED observed experimentally.

### 3.6. Recovery and recycling

#### 3.6.1. GA recovery

**Fig. 6:**
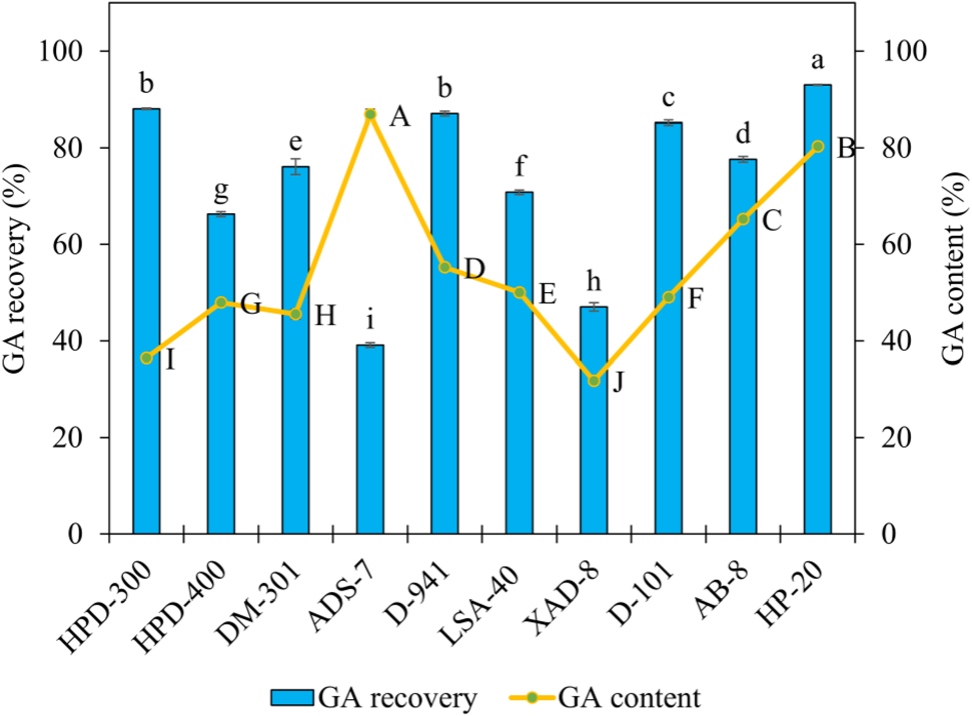
GA recovery and GA content obtained using different MRs in PED ABHS. Different letters indicate statistically significant differences among means (*p* < 0.05).

After obtaining GA enriched extracts from the PED ABHS, GA recovery was carried out using SLE approach based on MRs in order to evaluate the enrichment efficiency and selective recovery of the target compound. The recovery performance was assessed using two key parameters, namely GA recovery (%) and GA content (%) in the desorbed fraction, as presented in Fig. 6.

GA recovery exhibited a pronounced dependence on the surface properties and pore structure of the MRs. Among the tested MRs, HP-20 provided the highest recovery efficiency (92.98%) while maintaining a remarkably high GA content (80.31%). This behavior indicates favorable interactions between the moderately hydrophobic resin surface and the triterpenoid aglycone moiety of GA, while still allowing efficient desorption in the subsequent step. Other resins such as HPD-300, D-941, and D-101 also showed relatively high recovery efficiencies above 85%. However, their GA contents were substantially lower, suggesting limited selectivity or co-adsorption of polar impurities originating from the ABHS matrix. In contrast, MRs with higher polarity or restricted surface area, including ADS-7 and XAD-8,^42^ exhibited lower recovery efficiencies, highlighting their poor compatibility with the hydrogen bond-rich PED solvent environment.

The discrepancy between recovery efficiency and GA content highlights that the effectiveness of the SLE process is governed not only by the adsorption capacity toward GA but also by the selectivity of MRs relative to co extracted components in the ABHS. In this context, HP-20 offers an optimal balance between adsorption capacity, selectivity, and desorption efficiency, making it the most suitable material for GA recovery following extraction with PED ABHS.

In practical terms, these findings demonstrate that integrating ABHS based extraction with an MRs assisted SLE recovery step not only enhances GA extraction performance but also enables efficient recovery and enrichment of the target compound. This integrated strategy contributes to the development of a green extraction process by reducing reliance on conventional organic solvents and improving the feasibility of large scale applications.

#### 3.6.2. Reusability of ABHS and MRs

The reusability of the extraction and recovery system was systematically evaluated to assess its operational stability and suitability for repeated use. The reuse protocol involved consecutive extraction cycles employing regenerated PED ABHS, coupled with continuous GA recovery using regenerated macroporous resins. Three successive extraction–recovery cycles were performed, and performance was evaluated based on four key parameters: extraction yields, GA recovery, GA content, and ABHS recovery.

**Fig. 7:**
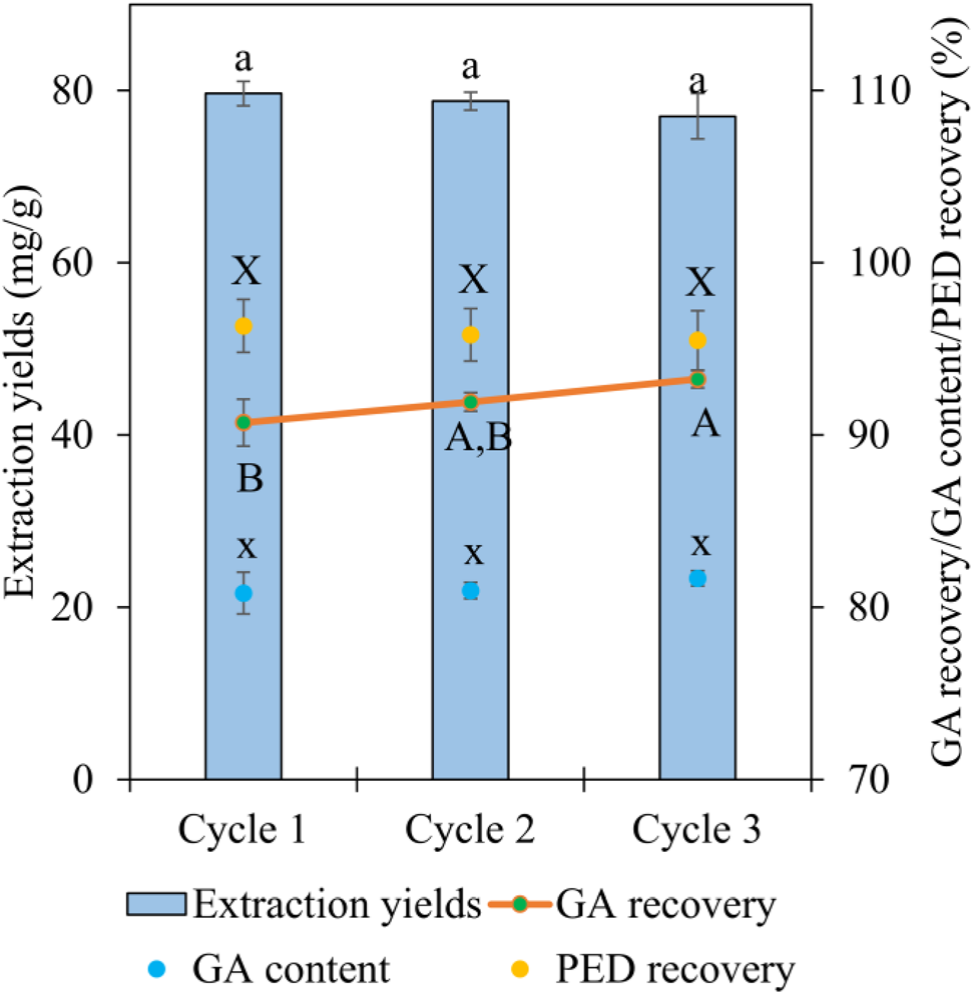
Reusability performance of PED ABHS and HP-20 MRs evaluated by extraction yields, GA recovery, GA content, and solvent recovery. Different letters indicate statistically significant differences among means (*p* < 0.05).

As summarized in Fig. 7 and the corresponding numerical data, the PED ABHS exhibited good recyclability over three consecutive cycles, with only marginal variations in extraction performance. The extraction yields remained virtually unchanged over three consecutive cycles, varying within a narrow range of 77.03 mg g^−1^ to 79.65 mg g^−1^ (*p* > 0.05). GA recovery showed only minor fluctuations between 90.72% and 93.25%, while GA content was consistently maintained at 80.82% to 81.67% (*p* > 0.05). Meanwhile, PED recovery consistently exceeded 95.50% across all cycles, reflecting negligible solvent loss and sustained phase stability during regeneration and reuse.

The structural integrity of GA after extraction using ABHS was comprehensively evaluated by combining UV spectral analysis obtained from HPLC-DAD and ^1^H NMR spectroscopy through direct comparison with the GA standard. The corresponding UV and ^1^H NMR spectra of the standard and extracted samples are presented in Fig. S6–S8.

Firstly, the UV spectra of the GA standard and the ABHS-extracted GA exhibited a high degree of similarity, with both samples showing a characteristic absorption maximum at approximately 254 nm. This result indicates that the chromophoric system of GA remained unchanged during the extraction process, suggesting the absence of structural degradation or transformation affecting its conjugated functional groups (Fig. S6).

Further structural confirmation was obtained from ^1^H NMR analysis. The spectra of both the GA standard and the ABHS-extracted sample displayed the key characteristic signals of GA. Specifically, seven methyl group signals were observed in the upfield region (*δ* 1.34–0.71 ppm), consistent with the pentacyclic triterpenoid framework. In addition, a distinct olefinic proton signal appeared at approximately *δ* 5.39 ppm in both samples, confirming the preservation of the unsaturated moiety. Notably, two anomeric proton signals at *δ* 4.49 and 4.41–4.40 ppm were clearly detected, indicating that the glycosidic units of GA remained intact after extraction (Fig. S7 and S8).

The consistent appearance of these diagnostic signals in both UV and ^1^H NMR spectra demonstrates that the fundamental structural features of GA, including both the aglycone and sugar moieties, were well preserved. Only minor variations in chemical shifts were observed, which can be reasonably attributed to differences in sample composition, GA content, and matrix effects within the extract, rather than any structural transformation. Overall, the combined spectroscopic evidence from UV and ^1^H NMR analyses provides strong support that the ABHS-based extraction process does not induce significant structural alteration of GA and effectively maintains its molecular integrity.

In summary, the combined reuse of ABHS and MRs demonstrates that the proposed extraction recovery strategy maintains high efficiency and selectivity over multiple cycles. The high solvent recovery rates, together with the stable extraction yield and GA recovery, underscore the potential of this integrated system for sustainable and large scale applications. These findings further strengthen the green credentials of the developed process by minimizing solvent consumption, reducing material waste, and enhancing process economy through effective reuse of both solvents and recovery media.

### 3.7. Evaluation of the anti-inflammatory activity of GA extracted using ABHS and organic solvents

In this study, ABHS have demonstrated considerable potential as a feasible and sustainable approach for the extraction of GA from licorice, with results indicating superior extraction efficiency compared to conventional organic solvents. Nevertheless, beyond extraction performance, it remains necessary to determine whether GA obtained using ABHS preserves its intrinsic biological properties or exhibits enhanced functional activity as a consequence of the extraction environment.

Anti-inflammatory activity was chosen as the primary biological endpoint because it represents the most characteristic and pharmacologically relevant activity of GA. The anti-inflammatory activity of GA extracted using the PED ABHS, denoted as GA–PED, was systematically compared with that of GA extracted using 50% EtOH, denoted as GA–EtOH. EtOH was selected as the reference organic solvent due to its relatively high extraction efficiency and its ability to produce extracts with elevated GA content among the tested organic solvents. The comparative evaluation was conducted through a two-step strategy, beginning with the assessment of NO production inhibition in an established *in vitro* inflammation model, followed by molecular docking analysis to further elucidate the mechanistic basis underlying the observed anti-inflammatory performance of the GA extracts.

#### 3.7.1. Inhibition of nitric oxide production

The anti-inflammatory activity of GA extracts obtained from the ABHS was evaluated based on their ability to inhibit NO production in an established *in vitro* inflammation model and was comparatively assessed against GA extracted with a conventional organic solvent. As presented in Table 3, all GA-containing extracts exhibited measurable NO inhibitory effects, with notable differences in potency depending on the extraction solvent.

**Table 3:** NO inhibitory activity of GA extracted with organic solvents and ABHS

Extract	IC_50_ values (µg mL^−1^)
GA–EtOH	67.86 ± 2.30
GA–PED	51.42 ± 1.64
Dexamethasone	11.93 ± 1.14

GA extracted using EtOH exhibited moderate inhibitory activity, with an IC_50_ value of 67.86 ± 2.30 µg mL^−1^. In contrast, GA recovered from the ABHS demonstrated significantly enhanced anti-inflammatory efficacy. The GA–PED extract showed a reduced IC_50_ value of 51.42 ± 1.64 µg mL^−1^, indicating improved NO inhibitory activity compared with the ethanolic extract. These findings suggest that ABHS extraction enhances the biological performance of GA relative to conventional ethanolic extraction.

The enhanced NO inhibitory activity observed for the GA–PED extract can be primarily attributed to the substantially higher GA content in the extract obtained using ABHS compared to the conventional organic solvent. Specifically, the GA–PED extract contained 80.31% GA, whereas the GA–EtOH extract exhibited a markedly lower GA content of only 25.28%. This pronounced enrichment of GA directly contributes to the improved anti-inflammatory efficacy observed for the ABHS-derived extract.

In addition to the increased GA content, the superior solubilization capacity of the ABHS and its ability to preserve the structural integrity of GA may further enhance biological performance. The unique solvent microenvironment provided by PED likely facilitates improved molecular stability and bioavailability of GA. Furthermore, the ABHS may promote the co-extraction of minor bioactive constituents that exert synergistic effects with GA, thereby contributing to the overall enhancement of anti-inflammatory activity. The reduced IC_50_ value observed for GA–PED compared with GA–EtOH further underscores the critical role of solvent polarity modulation and hydrotropic interactions in governing the biological efficacy of the extracted compound. Nevertheless, these interpretations remain preliminary and are mainly inferred from the experimental observations. Therefore, further in-depth investigations are required to elucidate the precise mechanistic pathways underlying this enhanced anti-inflammatory activity.

To further substantiate this assumption, the chemical profile of the GA–PED extract was characterized by UHPLC-Q-TOF-MS/MS, and the identified constituents are summarized in Table S6. In addition to GA, several minor compounds were detected, predominantly belonging to flavonoid and isoflavonoid classes, including liquiritin, isoliquiritin, liquiritigenin, isoliquiritigenin, formononetin, glabridin, and glabrol derivatives. These compounds are well-recognized constituents of licorice and have been reported to possess anti-inflammatory properties through multiple mechanisms.^43^ For example, chalcone and flavanone derivatives such as isoliquiritigenin and liquiritigenin are known to modulate inflammatory signaling pathways,^44^ while prenylated isoflavonoids like glabridin contribute to the attenuation of oxidative stress and inflammatory responses.^45^

The coexistence of these structurally diverse compounds in the GA–PED extract suggests that the observed NO inhibitory activity may not be exclusively attributed to GA, but could also be influenced by additive or synergistic interactions among co-extracted constituents. Such multi-component interactions are commonly observed in plant-derived extracts and may enhance biological efficacy through complementary modes of action. These findings provide supporting evidence for the contribution of minor components to the overall anti-inflammatory performance of the ABHS-derived extract.

#### 3.7.2. Molecular docking

To gain deeper mechanistic insight into the enhanced anti-inflammatory activity observed for the GA–PED extract, molecular docking analysis was performed to examine the binding interactions between GA and the inflammation-related target protein iNOS at the molecular level.

Molecular docking indicated favorable binding of GA to iNOS, with the best docking pose showing a binding energy of −10.2 kcal mol^−1^. This relatively low binding energy suggests that GA can form a stable complex within the active site of iNOS, supporting the experimentally observed reduction of NO levels in LPS-stimulated RAW 264.7 macrophages.

**Fig. 8:**
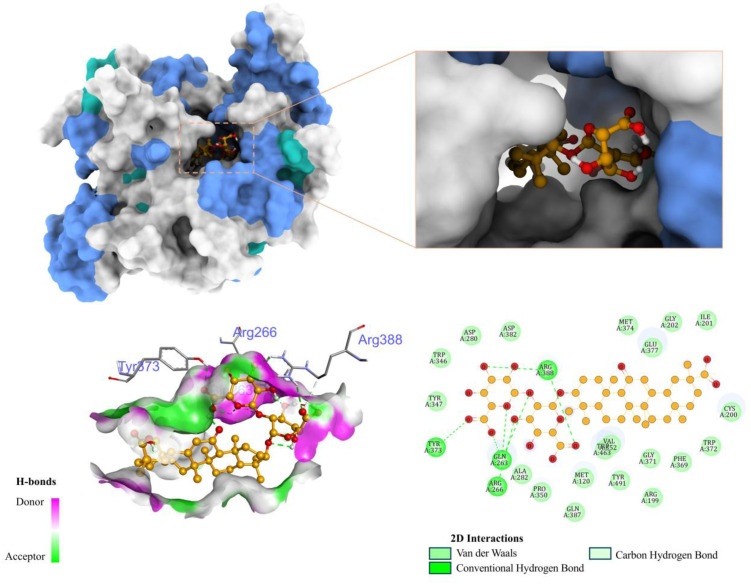
Structural visualization of GA docked into iNOS: overall binding pocket, active-site pose, hydrogen-bond surface mapping, and 2D interaction diagram.

The 3D interaction profile shows that GA fits deeply into the catalytic cavity near the heme center, where oxidation of l-arginine occurs (Fig. 8). The hydrophobic triterpenoid aglycone moiety occupies the non-polar core of the binding pocket, forming van der Waals interactions with Glu377, Cys200, Phe369, Ile201, and several adjacent residues. This positioning helps stabilize the ligand orientation and allows the highly polar glycone portion to extend toward the pocket entrance.

Within the glycone region, GA forms four hydrogen bonds with active-site residues, specifically Gln263, Arg266, Arg388, and Tyr373. Among these, Gln263, Arg266, and Arg388 are classified as first-shell residues of iNOS according to Tao *et al.*^46^ These first-shell residues are known to directly participate in the recognition and stabilization of iNOS inhibitors. The ability of GA to interact with most of these key residues reinforces the plausibility of the predicted binding pose and aligns well with previously reported interaction patterns.

In addition, complementary interactions between the hydroxyl and carboxyl groups of GA and the hydrogen-bond donor/acceptor residues within the active site further stabilize the predicted complex. Such interactions may interfere with substrate (l-arginine) access or disrupt critical conformational dynamics of the catalytic machinery, thereby reducing enzymatic activity.

In this context, the docking model illustrates that GA engages the iNOS active site through a combination of hydrophobic core interactions and peripheral hydrogen bonding. This binding mode is consistent with its observed NO-inhibitory activity *in vitro* and supports the hypothesis that iNOS represents a key molecular target contributing to the anti-inflammatory effects of GA.

### 3.8. Toxicity assessment

Although the GA-enriched fraction obtained from PED exhibited enhanced anti-inflammatory activity, a comprehensive evaluation of its cytotoxicity was necessary to ensure that the improved bioefficacy was not accompanied by adverse effects on normal human cells. In addition, the crude PED extract was also assessed to determine whether the extraction matrix introduced any cytotoxic components. The cytotoxicity assays were performed using HEK-293A cells, a human embryonic kidney cell line widely employed as a representative non-cancerous model for preliminary toxicity screening.

As summarized in Table 4, both the crude PED extract (Ext–PED) and the GA-enriched fraction (GA–PED) exhibited negligible cytotoxicity toward HEK-293A cells, with IC_50_ values exceeding 100 µg mL^−1^. These results indicate that neither the extraction matrix nor the enrichment process introduced additional cytotoxic constituents. Collectively, the findings suggest that the PED-based extraction and recovery strategy preserves the biological safety profile of GA under the investigated conditions, thereby supporting its suitability for subsequent bioactivity evaluation and potential therapeutic applications.

**Table 4:** Cytotoxicity of crude PED extract and GA-enriched fraction on HEK-293A cells

Extract	IC_50_ values (µg mL^−1^)
Ext-PED^a^	>100
GA–PED^b^	>100
Ellipticine	0.31 ± 0.02

a Crude extract obtained using PED ABHS.

b GA-enriched fraction recovered from PED ABHS using MRs.

Beyond the demonstrated extraction efficiency, biological activity, and favorable safety profile, the practical scalability of the PED-based ABHS system is also of considerable interest. PED is a bio-based solvent with relatively low toxicity and increasing commercial availability, making it suitable for large-scale applications.^20^ In addition, ultrasound-assisted extraction can be readily scaled up using industrial ultrasonic reactors, which have been widely implemented in natural product processing.^47^ The use of MRs further enhances process feasibility, as these materials can be efficiently regenerated and reused over multiple cycles, thereby reducing operational costs and solvent consumption.^48^ Collectively, these features highlight the practical potential of the proposed system for industrial-scale extraction of GA.

## 4. Conclusion

This work demonstrates the effectiveness of ABHS as a sustainable solvent platform for extracting GA from licorice. Among the investigated hydrotropes, PED showed the best extraction performance. Under optimized conditions (67.9% ABHS, liquid–solid ratio 49.9 mL g^−1^, 17.0 min, 70 °C), the GA yield reached 79.43 ± 1.13 mg g^−1^, which was markedly higher than that obtained using aqueous alcohols (≈49 mg g^−1^) and reported DES systems (45.47–61.29 mg g^−1^). Efficient recovery of GA was achieved using the HP-20 MRs, providing 92.98% recovery with 80.31% purity, while the solvent system maintained stable extraction performance and solvent recovery above 95.50% over three reuse cycles. Kinetic analysis indicated a diffusion-controlled extraction process with an activation energy of 19.84 kJ mol^−1^. Quantum chemical calculations revealed stronger interactions between GA and PED (−19.798 kcal mol^−1^) compared with water (−12.137 kcal mol^−1^) and EtOH (−15.307 kcal mol^−1^), explaining the improved extraction efficiency. Furthermore, the GA-enriched fraction obtained using PED showed enhanced anti-inflammatory activity (IC_50_ = 51.42 ± 1.64 µg mL^−1^) compared with the ethanolic extract (67.86 ± 2.30 µg mL^−1^) without detectable cytotoxicity toward HEK-293A cells (IC_50_ > 100 µg mL^−1^). Molecular docking further supported the interaction of GA with the iNOS active site with a binding affinity of −10.2 kcal mol^−1^. Overall, these findings establish ABHS as efficient, recyclable, and mechanistically well-understood solvents for GA extraction. The integrated experimental and computational framework presented here provides a rational basis for designing green extraction systems and may be extended to other amphiphilic triterpenoid saponins and related bioactive compounds. Future studies may systematically evaluate the applicability of this ABHS platform across a broader range of structurally diverse saponins, including ginsenosides and saikosaponins, to further validate its generality and expand its potential in natural product processing.

## Author contributions

Nhan Trong Le: methodology, investigation, writing – original draft preparation; Hang Thanh Thi Phan: investigation, formal analysis; The-Huan Tran: software, writing – review and editing; Hung The Nguyen: formal analysis, writing – review and editing; Hoai Thi Nguyen: conceptualization, methodology, writing – review and editing, supervision.

## Conflicts of interest

There are no conflicts to declare.

## Supplementary Material

RA-016-D6RA02244H-s001

## Data Availability

Data will be made available upon request. Supplementary information (SI) is available. See DOI: https://doi.org/10.1039/d6ra02244h.

## References

[cit1] Park Y.-S., Kang S.-M., Kim Y.-J., Lee I.-J. (2024). J. Ethnopharmacol..

[cit2] Jiang L., Akram W., Luo B., Hu S., Faruque M. O., Ahmad S., Yasin N. A., Khan W. U., Ahmad A., Shikov A. N., Chen J., Hu X. (2021). Front. Pharmacol.

[cit3] Selyutina O. Y., Polyakov N. E. (2019). Int. J. Pharm..

[cit4] Wang L., Chen X., Liu J., Tan Z. (2021). J. Mol. Liq..

[cit5] Lanjekar K. J., Rathod V. K. (2021). Process Biochem..

[cit6] Pandit B., Saha P. (2025). Sustain. Chem. Clim. Act..

[cit7] Maki M. A. A., Tan K. F., Yee M. T. S., Mishra D. K., Venkatesh M. P., Abduljaleel O. W., Karahan M., Palanirajan V. K. (2025). Discover Chem..

[cit8] tul Kubra K., Ahmed D., Aydar A. Y., Qamar M. T. (2023). Sustainable Chem. Pharm..

[cit9] Dong J., Wu G., Dong Z., Yang D., Bo Y., An M., Zhao L. (2021). RSC Adv..

[cit10] Lanjekar K. J., Rathod V. K. (2021). Ind. Eng. Chem. Res..

[cit11] Yu P., Li Q., Feng Y., Ma S., Chen Y., Li G. (2021). Molecules.

[cit12] Lanjekar K. J., Rathod V. K. (2024). Prep. Biochem. Biotechnol..

[cit13] Yahaya N., Mohamed A. H., Sajid M., Zain N. N. M., Liao P.-C., Chew K. W. (2024). Carbohydr. Polym..

[cit14] Rahman A. M. A., Bakar A. R. A., Yee A. Q., Zainudin M. A. M., Daud N. M. A. N., Gunny A. A. N., Sarip M. S. M., Peron R. V., Khairuddin N. H. (2025). RSC Adv..

[cit15] Yue Y., Huang Q., Fu Y., Chang J. (2020). RSC Adv..

[cit16] Alias A. H. D., Shafie M. H. (2025). Microchem. J..

[cit17] Nasrallah S., Minceva M. (2025). Mol. Pharm..

[cit18] Abranches D. O., Soares B. P., Ferreira A. M., Shimizu S., Pinho S. P., Coutinho J. A. P. (2022). Phys. Chem. Chem. Phys..

[cit19] Zakharova L. Y., Vasilieva E. A., Mirgorodskaya A. B., V Zakharov S., V Pavlov R., Kashapova N. E., Gaynanova G. A. (2023). J. Mol. Liq..

[cit20] Le N. T., Nguyen L. T., Thi Nguyen N. A., Nguyen H. T. (2025). Sep. Purif. Technol..

[cit21] Soares B. P., Abranches D. O., Sintra T. E., Leal-Duaso A., García J. I., Pires E., Shimizu S., Pinho S. P., Coutinho J. A. P. (2020). ACS Sustain. Chem. Eng..

[cit22] Silva S. S., Abranches D. O., Pinto A. S., Soares B. P., Passos H., Ferreira A. M., Coutinho J. A. P. (2023). Ind. Eng. Chem. Res..

[cit23] Vieira V., Calhelha R. C., Barros L., Coutinho J. A. P., Ferreira I. C. F. R., Ferreira O. (2020). Molecules.

[cit24] Soares B. P., Ferreira A. M., Justi M., Rodrigues L. G., V Oliveira J., Pinho S. P., Coutinho J. A. P. (2023). Molecules.

[cit25] Baky M. H., Elsaid M. B., Farag M. A. (2022). Phytochemistry.

[cit26] Vietnamese Pharmacopoeia V. , Medical Publishing House, Hanoi, Vietnam, 2017

[cit27] Neese F. (2025). Wiley Interdiscip. Rev.: Comput. Mol. Sci..

[cit28] Neese F. (2003). J. Comput. Chem..

[cit29] Garcia-Ratés M., Neese F. (2020). J. Comput. Chem..

[cit30] Neese F. (2023). J. Comput. Chem..

[cit31] Hanwell M. D., Curtis D. E., Lonie D. C., Vandermeersch T., Zurek E., Hutchison G. R. (2012). J. Cheminf..

[cit32] Lu T., Chen Q. (2021). Chem.:Methods.

[cit33] Lu T., Chen F. (2012). J. Comput. Chem..

[cit34] Lu T. (2024). J. Chem. Phys..

[cit35] Humphrey W., Dalke A., Schulten K. (1996). J. Mol. Graphics.

[cit36] Trott O., Olson A. J. (2010). J. Comput. Chem..

[cit37] Garcin E. D., Arvai A. S., Rosenfeld R. J., Kroeger M. D., Crane B. R., Andersson G., Andrews G., Hamley P. J., Mallinder P. R., Nicholls D. J., St-Gallay S. A., Tinker A. C., Gensmantel N. P., Mete A., Cheshire D. R., Connolly S., Stuehr D. J., Åberg A., V Wallace A., Tainer J. A., Getzoff E. D. (2008). Nat. Chem. Biol..

[cit38] Hoang T. H. X., Tran T.-H., Doan T. Q., Le A. T., Nguyen H. T., Tran L. T. T. (2025). Chem. Biodiversity.

[cit39] Meng E. C., Goddard T. D., Pettersen E. F., Couch G. S., Pearson Z. J., Morris J. H., Ferrin T. E. (2023). Protein Sci..

[cit40] Tsai P. J., Tsai T. H., Yu C. H., Ho S. C. (2007). Food Chem..

[cit41] Skehan P., Storeng R., Scudiero D., Monks A., McMahon J., Vistica D., Warren J., Bokesch H., Kenney S., Boyd M. (1990). J. Natl. Cancer Inst..

[cit42] Le N. T., Ho H. T. T., Duong T. D., Le T. T., Nguyen Q. P., Nguyen T. N. T., Nguyen H. T. (2024). Sep.
Sci. Technol..

[cit43] Dang L., Jin Y., Yuan Y., Shao R., Wang Y., Herb A. (2024). Med..

[cit44] Ramalingam M., Kim H., Lee Y., Lee Y.-I. (2018). Front. Aging Neurosci..

[cit45] Simmler C., Pauli G. F., Chen S.-N. (2013). Fitoterapia.

[cit46] Tao Y., Yang S., Xu H., Tao X. (2019). SN Appl. Sci..

[cit47] Shen L., Pang S., Zhong M., Sun Y., Qayum A., Liu Y., Rashid A., Xu B., Liang Q., Ma H., Ren X. (2023). Ultrason. Sonochem..

[cit48] Della Posta S., Gallo V., Gentili A., Fanali C. (2022). TrAC, Trends Anal. Chem..

