# Supplementary material for: Aqueous bio-based hydrotropic solutions for green extraction of glycyrrhizic acid from licorice: experimental investigation and mechanistic insights
Source: RSC Adv. 2026 Apr 21;16(23):20866–82. doi: 10.1039/d6ra02244h (PMC13097309; doi:10.1039/d6ra02244h)
Supplement: RA-016-D6RA02244H-s001 [file RA-016-D6RA02244H-s001.pdf]

Supplementary Information (SI) for RSC Advances.
This journal is © The Royal Society of Chemistry 2026

Aqueous Bio-Based Hydrotropic Solutions for Green Extraction of Glycyrrhizic Acid from Licorice: Experimental Investigation and Mechanistic Insights

Nhan Trong Le,[a] Hang Thanh Thi Phan,[a] The-Huan Tran[a], Hung The Nguyen,[b] and Hoai Thi Nguyen*[a]

[a] Hue University of Medicine and Pharmacy, Hue University, Hue City, Vietnam.

[b] Hanoi University of Pharmacy, Hanoi City, Vietnam.

* Correspondence: Hoai Thi Nguyen; e-mail: nthoai@hueuni.edu.vn; Hue University of Medicine and Pharmacy, Hue University, Hue City, Vietnam.

Supplementary material contents

- **Number of pages: 10**
- **Number of tables: 6**
- **Number of figures: 8**

Table S1: Independent variables and their coded levels in the BBD

Independent variables	Coded symbols	Coded levels		
		**-1**	**0**	**+1**
ABHS concentration (%)	A	25	50	75
Liquid-solid ratios (mL/g)	B	20	35	50
Extraction time (min)	C	10	35	60
Extraction temperature (°C)	D	30	50	70

Table S2. BBD results for the extraction yields of GA using PED ABHS

Run	A	B	C	D	Extraction efficiency (mg/g)
1	50	20	35	70	69.86
2	50	35	35	50	73.71
3	50	35	60	30	71.62
4	75	35	60	50	72.80
5	50	35	10	70	76.01
6	50	20	60	50	67.51
7	25	35	60	50	71.99
8	50	50	35	70	78.30
9	25	35	35	30	70.82
10	25	50	35	50	75.16
11	75	35	10	50	72.21
12	75	50	35	50	76.86
13	50	20	10	50	66.08
14	75	35	35	30	70.96
15	75	35	35	70	74.92
16	75	20	35	50	68.13
17	50	50	10	50	75.32
18	50	20	35	30	65.74
19	25	20	35	50	66.95
20	50	35	35	50	72.60
21	50	35	35	50	72.25
22	50	50	35	30	74.44
23	50	35	35	50	72.10
24	50	35	10	30	68.17
25	50	50	60	50	74.53
26	50	35	35	50	72.35
27	25	35	35	70	74.32
28	50	35	60	70	72.66
29	25	35	10	50	70.69

A: ABHS concentration, **B**: liquid-solid ratio, **C**: extraction time, and **D**: extraction temperature

Table S3. ANOVA results for GA extraction using the PED ABHS

Source	Sum of Squares	df	Mean Square	F-value	p-value	
Model	286.44	14	20.46	88.67	< 0.0001	significant
A	2.95	1	2.95	12.80	0.0030	
B	211.16	1	211.16	915.16	< 0.0001	
C	0.5728	1	0.5728	2.48	0.1374	
D	49.28	1	49.28	213.59	< 0.0001	
AB	0.0682	1	0.0682	0.2954	0.5953	
AC	0.1253	1	0.1253	0.5428	0.4734	
AD	0.0511	1	0.0511	0.2213	0.6453	
BC	1.23	1	1.23	5.34	0.0365	
BD	0.0170	1	0.0170	0.0735	0.7903	
CD	11.58	1	11.58	50.19	< 0.0001	
A^2	0.0002	1	0.0002	0.0008	0.9783	
B^2	4.78	1	4.78	20.72	0.0005	
C^2	3.87	1	3.87	16.75	0.0011	
D^2	0.4329	1	0.4329	1.88	0.1923	
Residual	3.23	14	0.2307			
Lack of Fit	1.57	10	0.1571	0.3789	0.9028	not significant
Pure Error	1.66	4	0.4147			
Cor Total	289.67	28				

$R^2 = 0.9888$ R^2 adj = 0.9777 R^2 pred = 0.9598

A: ABHS concentration, **B**: liquid-solid ratio, **C**: extraction time, and **D**: extraction temperature

Table S4. The relative deviations between the experimental results and model predictions in the trials of extracting GA from Licorice using PED ABHS

Run	A	B	C	D	Extraction yield (mg/g)	Relative deviation

					Experimental	Predicted	(%)
1	50	20	35	70	69.86	69.90	-0.06
2	50	35	35	50	73.71	72.60	1.53
3	50	35	60	30	71.62	71.98	-0.50
4	75	35	60	50	72.80	72.37	0.59
5	50	35	10	70	76.01	75.60	0.54
6	50	20	60	50	67.51	67.55	-0.06
7	25	35	60	50	71.99	71.74	0.35
8	50	50	35	70	78.30	78.16	0.18
9	25	35	35	30	70.82	70.46	0.51
10	25	50	35	50	75.16	75.32	-0.21
11	75	35	10	50	72.21	72.29	-0.11
12	75	50	35	50	76.86	76.57	0.38
13	50	20	10	50	66.08	66.00	0.12
14	75	35	35	30	70.96	71.22	-0.37
15	75	35	35	70	74.92	75.50	-0.77
16	75	20	35	50	68.13	67.92	0.31
17	50	50	10	50	75.32	75.50	-0.24
18	50	20	35	30	65.74	65.72	0.03
19	25	20	35	50	66.95	67.19	-0.36
20	50	35	35	50	72.60	72.60	0.00
21	50	35	35	50	72.25	72.60	-0.48
22	50	50	35	30	74.44	74.24	0.27
23	50	35	35	50	72.10	72.60	-0.69
24	50	35	10	30	68.17	68.14	0.04
25	50	50	60	50	74.53	74.83	-0.40
26	50	35	35	50	72.35	72.60	-0.34
27	25	35	35	70	74.32	74.28	0.05
28	50	35	60	70	72.66	72.63	0.04
29	25	35	10	50	70.69	70.94	-0.35

A: ABHS concentration, **B**: liquid-solid ratio, **C**: extraction time, and **D**: extraction temperature

Table S5. Abbreviations, compositions, and extraction conditions of solvents employed for the comparative evaluation of GA extraction from Licorice.

No.	Abbreviation	Composition of solvent	Extraction conditions	Ref.
1	DESs-1	Glycerol-ammonium acetate (3:1 mol/mol), C = 50%	L/S ratio 24 mL/g, 25 min, 80 °C, HAE	1
2	DESs-2	Glycerol-ammonium acetate (3:1 mol/mol), C = 50%	L/S ratio 24 mL/g, 79 min, 60 °C, UAE	1
3	DESs-3	Glycerol-choline chloride (3:1 mol/mol), C = 50%	L/S ratio 24 mL/g, 65 min, 80 °C, HAE	1
4	DESs-4	Glycerol-choline chloride (3:1 mol/mol), C = 50%	L/S ratio 24 mL/g, 25 min, 80 °C, UAE	1
5	DESs-5	Choline chloride-lactic acid (1:1 mol/mol), C = 70%	L/S ratio 40 mL/g, 30 min, 40 °C, HAE	2
6	DESs-6	Choline chloride-lactic acid (1:1 mol/mol), C = 70%	L/S ratio 30 mL/g, 15 min, room temperature, UAE	3
7	DESs-7	Choline chloride-1,3-butanediol (1:4 mol/mol), C = 70%	L/S ratio 20 mL/g, 41 min, 50 °C, UAE	4
8	DESs-8	1,4-butanediol–levulinic acid (1:3 mol/mol), C = 83%	L/S ratio 42 mL/g, 30 min, room temperature, UAE	5

L/S ratio: the liquid-solid ratio; UAE: ultrasound-assisted extraction; HAE: heat-assisted extraction

Table S6: Chemical composition of the GA-enriched extract obtained from Licorice using ABHS

No	RT (min)	Identification	Elemental composition	Proposed ions	Experimental mass m/z	Theoretical mass m/z	Mass error (ppm)
1	7.70	Liquiritigenin	$C_{15}H_{12}O_4$	$[M-H]^-$	255.0653	255.0658	1.7990
2	7.70	Liquiritin	$C_{21}H_{22}O_9$	$[M-H]^-$	417.1168	417.1186	4.2728
3	8.92	Isoliquiritin	$C_{21}H_{22}O_9$	$[M-H]^-$	417.1173	417.1186	3.0741
4	9.03	Isoliquiritigenin	$C_{15}H_{12}O_4$	$[M-H]^-$	255.0656	255.0658	0.6234
5	10.30	Echinatin	$C_{16}H_{14}O_4$	$[M-H]^-$	269.0814	269.0814	0.0331
6	11.15	Formononetin	$C_{16}H_{12}O_4$	$[M-H]^-$	267.0659	267.0658	0.5285
7	11.22	Glycyrrhizic acid	$C_{42}H_{62}O_{16}$	$[M-H]^-$	821.3975	821.3960	1.8262
8	12.41	Licoisoflavanone	$C_{20}H_{18}O_6$	$[M-H]^-$	353.1018	353.1025	2.0909
9	12.49	Glycycoumarin	$C_{21}H_{20}O_6$	$[M-H]^-$	367.1168	367.1182	3.7818
10	12.78	Glabrene	$C_{20}H_{18}O_4$	$[M-H]^-$	321.1120	321.1127	2.2081
11	13.35	Glycyrol	$C_{21}H_{18}O_6$	$[M-H]^-$	365.1022	365.1022	0.0000
12	13.71	Glabridin	$C_{20}H_{20}O_4$	$[M-H]^-$	323.1276	323.1284	2.3493
13	13.77	Glycyrrhisoflavone	$C_{20}H_{18}O_6$	$[M-H]^-$	353.1022	353.1025	0.9581
14	14.35	Glabrone	$C_{20}H_{16}O_5$	$[M-H]^-$	335.0915	335.092	1.4134
15	14.40	Glabrol	$C_{25}H_{28}O_4$	$[M-H]^-$	391.1891	391.1910	4.7531
16	14.97	3-Hydroxyglabrol	$C_{25}H_{28}O_5$	$[M-H]^-$	407.1841	407.1859	4.3567
17	15.92	Licocoumarin A	$C_{25}H_{26}O_5$	$[M-H]^-$	405.1689	405.1702	3.2676

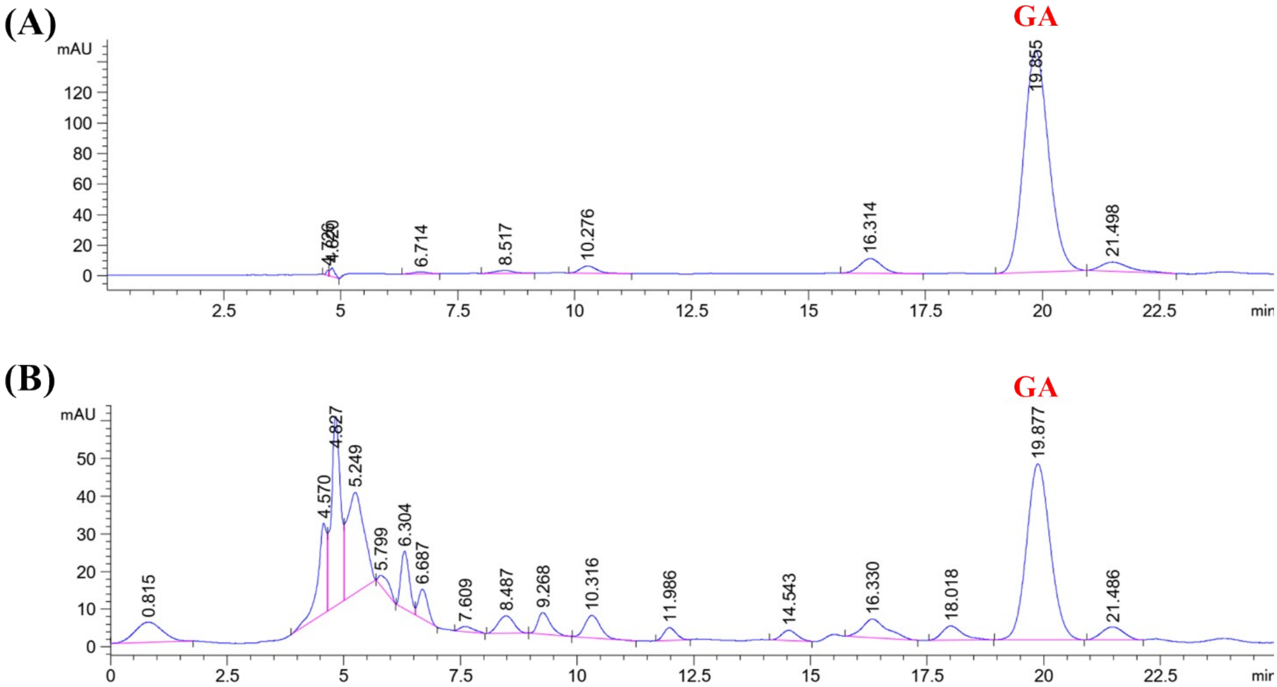

Figure S1. HPLC chromatograms for identification of GA at 254 nm. (A) reference standard (GA) and (B) Licorice extract.

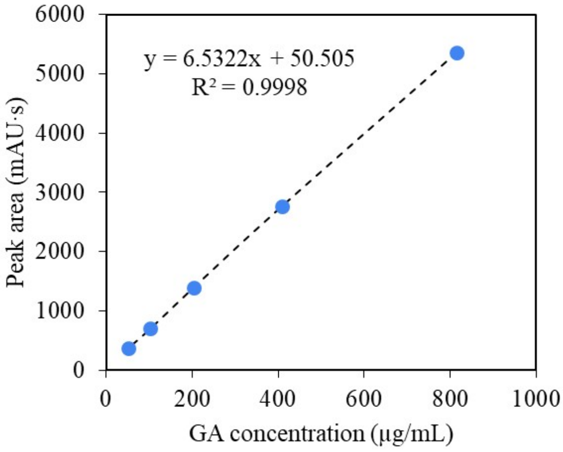

Figure S2. Calibration curve of GA showing the relationship between peak area and concentration ($R^2 = 0.9998$).

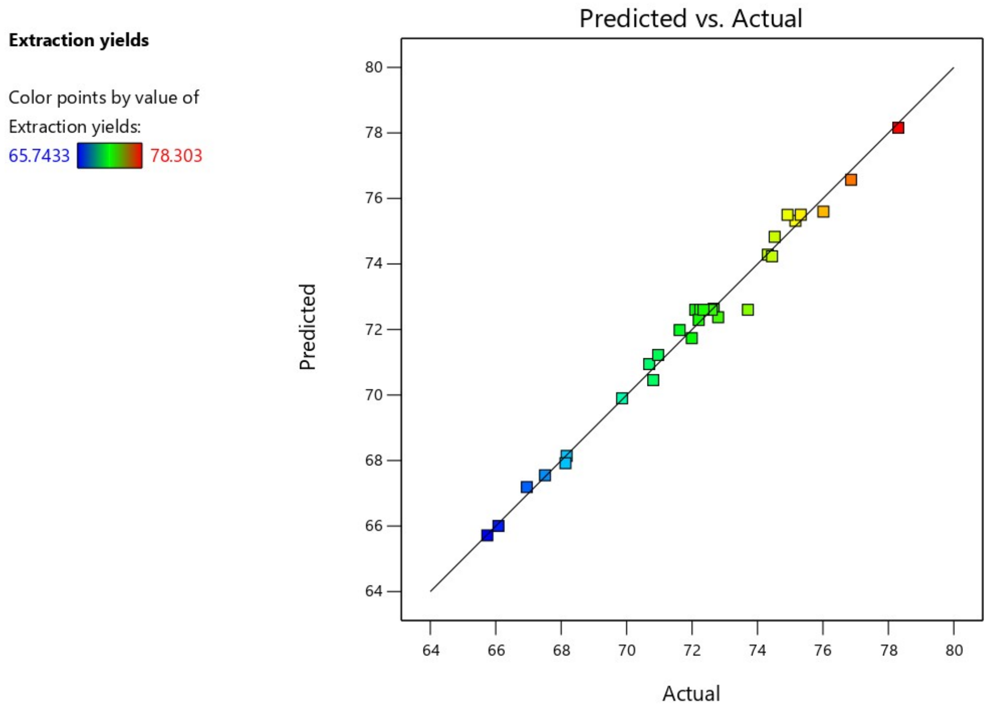

Figure S3. Plot of predicted versus observed values for GA extraction from Licorice using PED ABHS.

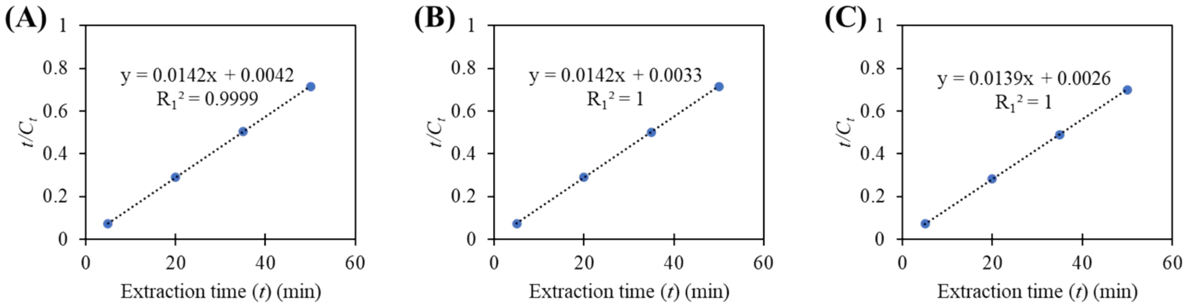

Figure S4: Second-order kinetic models for the GA extraction from Licorice using PED ABHS at different temperatures: A (40°C), B (50°C), and C (60°C).

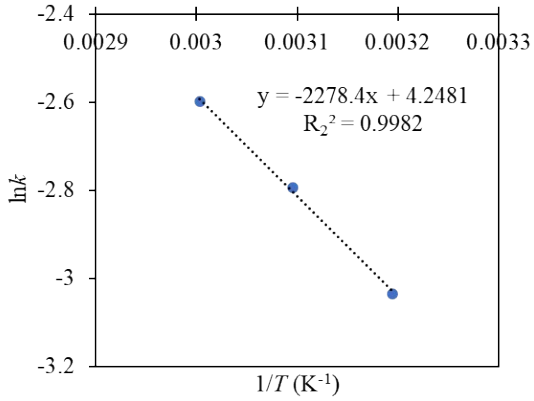

Figure S5: The Arrhenius plot of the second-order kinetic models for GA extraction from Licorice using PED ABHS.

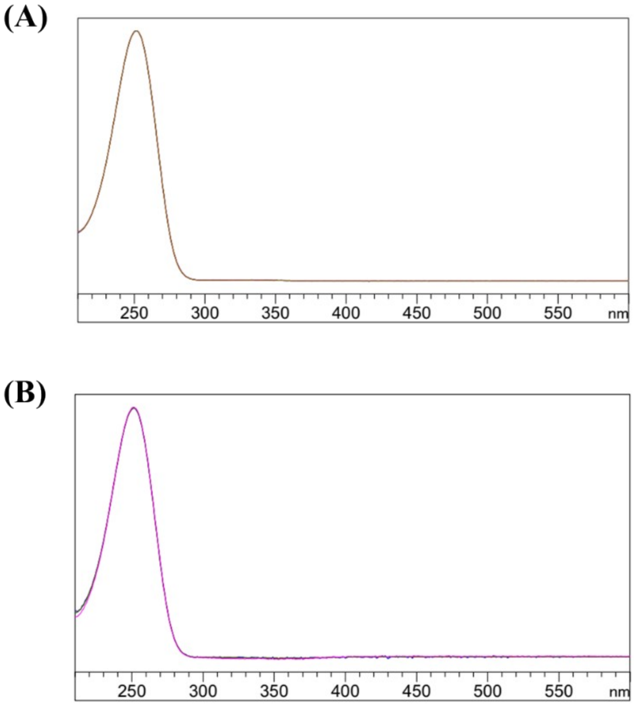

Figure S6. UV spectra GA standard (A) and ABHS-extracted GA (B).

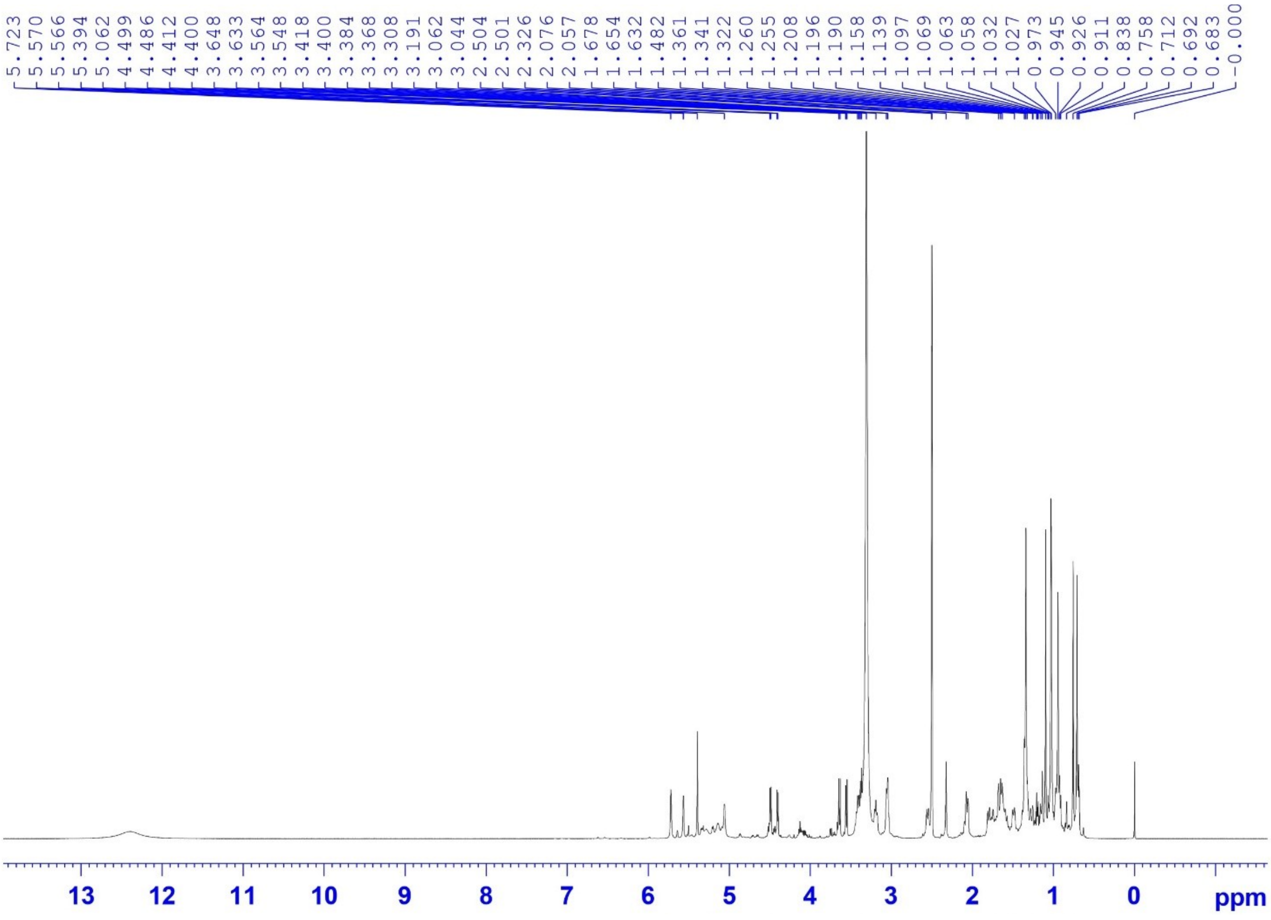

Figure S7. ^{1}H NMR spectra of standard GA.

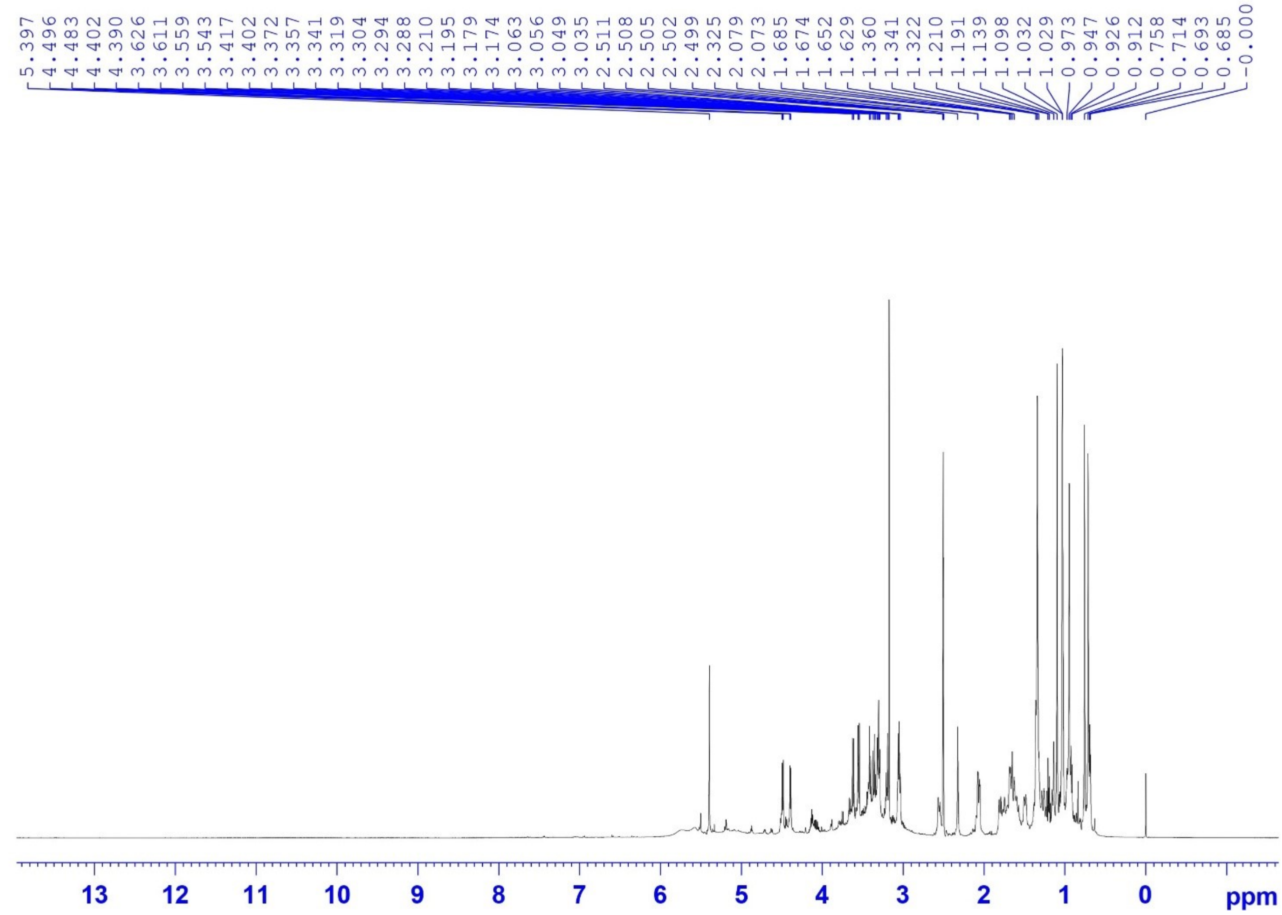

Figure S8. ^{1}H NMR spectra of ABHS-extracted GA

References

1 K. tul Kubra, D. Ahmed, A. Y. Aydar and M. T. Qamar, *Sustain. Chem. Pharm.*, 2023, **31**, 100910.
2 K. J. Lanjekar and V. K. Rathod, *Process Biochem.*, 2021, **102**, 22–32.
3 K. J. Lanjekar and V. K. Rathod, *Ind. Eng. Chem. Res.*, 2021, **60**, 9532–9538.
4 P. Yu, Q. Li, Y. Feng, S. Ma, Y. Chen and G. Li, *Molecules*, 2021, **26**, 1310.
5 J. Dong, G. Wu, Z. Dong, D. Yang, Y. Bo, M. An and L. Zhao, *RSC Adv.*, 2021, **11**, 37649–37660.
